# A multitaxa approach to biodiversity inventory in Matela protected area (Terceira, Azores, Portugal)

**DOI:** 10.3897/BDJ.12.e121884

**Published:** 2024-04-08

**Authors:** Mariana A. Sousa, Lucas Lamelas-López, Rui B. Elias, Rosalina Gabriel, Paulo A. V. Borges

**Affiliations:** 1 Mestrado em Gestão e Conservação da Natureza, University of the Azores Rua Capitão João d´Ávila, Pico da Urze 9700-042, Angra do Heroísmo, Azores, Portugal Mestrado em Gestão e Conservação da Natureza, University of the Azores Rua Capitão João d´Ávila, Pico da Urze 9700-042 Angra do Heroísmo, Azores Portugal; 2 cE3c- Centre for Ecology, Evolution and Environmental Changes/Azorean Biodiversity Group, CHANGE – Global Change and Sustainability Institute, School of Agricultural and Environmental Sciences, University of the Azores, Rua Capitão João d´Ávila, Pico da Urze, 9700-042, Angra do Heroísmo, Azores, Portugal cE3c- Centre for Ecology, Evolution and Environmental Changes/Azorean Biodiversity Group, CHANGE – Global Change and Sustainability Institute, School of Agricultural and Environmental Sciences, University of the Azores, Rua Capitão João d´Ávila, Pico da Urze, 9700-042 Angra do Heroísmo, Azores Portugal; 3 IUCN SSC Atlantic Islands Invertebrate Specialist Group, 9700-042, Angra do Heroísmo, Azores, Portugal IUCN SSC Atlantic Islands Invertebrate Specialist Group, 9700-042 Angra do Heroísmo, Azores Portugal; 4 IUCN SSC Species Monitoring Specialist Group, 9700-042, Angra do Heroísmo, Azores, Portugal IUCN SSC Species Monitoring Specialist Group, 9700-042 Angra do Heroísmo, Azores Portugal

**Keywords:** bryophytes, vascular plants, arthropods, birds, mammals, endemic species, introduced species, historical data, protected areas, Azores

## Abstract

**Background:**

This manuscript is the first contribution of the project, “Matela – uma ilha de biodiversidade” (“Matela - an island of biodiversity”), that aims to restore the native vegetation within the Azorean Protected Area of the Terceira Island Nature Park known as the "Protected Area for the Management of Habitats or Species of Matela" (TER08), situated on Terceira Island, the Azores Archipelago, Portugal. This small fragment of native forest, positioned at a low-medium altitude (300-400 m a.s.l.), is facing some conservation impacts as a consequence of the spread of different invasive exotic plant species, mainly *Pittosporumundulatum*, *Rubusulmifolius* and *Hedychiumgardnerianum*. The database we present encompasses diverse taxonomic groups, including bryophytes, vascular plants, arthropods, birds and mammals. It is derived from intensive sampling campaigns conducted in 2022, but some data from a previous vascular plant survey in 2015 were also included. The objective of this study was to provide an updated inventory of bryophytes, vascular plants, arthropods, birds and mammals within this protected area. In this way we are providing the reference conditions necessary for the monitoring of the impacts of the current ongoing restoration efforts within the project “Matela - an island of biodiversity”. Whenever feasible, the present inventory is juxtaposed with historical data from previous surveys conducted in Matela.

**New information:**

In the realm of bryophytes, our analysis revealed the presence of 75 taxa, comprising 44 mosses and 32 liverworts. Amongst these, 71 were indigenous, while three remained indeterminate and one, *Campylopusintroflexus*, was identified as invasive. A comparison with previous historical data revealed a decrease in species richness, which was partially counterbalanced by the discovery of 23 new recorded species in the area.

Regarding vascular plants, we distinguished 54 species, comprising 28 indigenous and 26 introduced taxa. Almost 80% of the inventoried species (n = 43) were newly documented in Matela.

The study of arthropods encompassed a total of 103 taxa. Within the realm of soil arthropods, we documented eight indigenous and 25 introduced taxa, witnessing the disappearance of endemic species alongside a substantial increase in introduced ones between 2002 and 2022. Canopy arthropods, totalling 36 indigenous and 18 introduced taxa, exhibited few changes when compared with data from 2002. SLAM traps captured 24 indigenous and 15 introduced arthropod taxa and no historical data are available for comparison.

As for avian species, we noted 12 indigenous birds and one introduced species, confirming the presence of most of the historical recorded native species.

The mammalian census revealed eight introduced species, setting new precedents for Matela, alongside the identification of one endemic species: the Azorean endemic bat *Nyctalusazoreum*.

## Introduction

The primary threats to biodiversity include biological invasions, climate change and habitat transformation and loss (e.g. Bellard et al. (2022)), all of which with dramatic impacts on native biota and ecosystems, profoundly altering ecological dynamics. The escalating magnitude of these threats has emerged as a paramount concern for scientists and conservation organisations, as highlighted by some seminal works (e.g. Simberloff et al. (2013), Bellard et al. (2016)), particularly on islands (Hanski 2016).

Protected areas play a central role in global commitments to sustainability and biodiversity conservation (Mulongoy and Chape 2004, Gaston et al. 2008). These areas were established to conserve ecosystems of recognised value, encompassing both fauna and flora, including habitats of high rarity and integrity (Mulongoy and Chape 2004, Gaston et al. 2008), harbouring complex communities of native and endemic species, acting as natural buffers against climate change and functioning as genetic reservoirs, fulfilling numerous other roles (e.g. Mulongoy and Chape (2004)). In this sense, several studies have been developed to evaluate the best species distribution models, under current and future climate conditions and to assess the effectiveness of protected areas to represent biodiversity (e.g. Ferreira et al. (2016), Vergílio et al. (2016), Silva et al. (2017), Ferreira et al. (2019)).

There are recommendations from the scientific community and government entities to increase the surface area of protected areas worldwide from 10-12% of the total surface of the planet (Gaston et al. 2008), to close to 50% (Wilson 2016 - Half-Earth Project). For instance, the European Union's Biodiversity Strategy for 2030 aims to transform at least 30% of Europe's land and seas into effectively managed protected areas, complementing the Natura 2000 network areas (European-Commission 2021).

Conserving biodiversity poses distinct challenges in ecologically sensitive regions such as islands (Borges et al. 2018), where endemism rates are high and species often exhibit low abundance or occupy extremely restricted areas. For example, in the Azores Archipelago, native biodiversity is particularly vulnerable to the spread of invasive species (Hortal et al. 2010, Lourenço et al. 2011), habitat fragmentation (Vergílio et al. 2016) or climate change impacts (Ferreira et al. 2016, Patiño et al. 2016).

Since the 1980s, the Azorean government established protected areas for biodiversity conservation, encompassing terrestrial habitats, coastal zones and mountain areas, as well as a considerable number of protected habitats, such as coastal shrubland, heathlands and Laurissilva forests. The Azores Protected Areas Network includes nine Island Natural Parks (one for each island), with a total of 124 protected areas, comprising 19 Natural Reserves, 11 Natural Monuments, 48 Protected Areas for Habitat or Species Management, 16 Protected Landscape Areas and 30 Protected Areas for Resource Management (Regional-Directorate-for-the-Environment 2020). This accounts for a total of 180,374 hectares of protected areas, equivalent to approximately 21% of the terrestrial area of the Archipelago. Of this, 56,219 hectares are terrestrial, while 124,155 hectares are marine (Regional-Directorate-for-the-Environment 2020).

Specifically, on Terceira Island, the Natural Park currently comprises 20 protected areas, including three Natural Reserves, two Natural Monuments, seven Protected Areas for Habitat or Species Management, one Protected Landscape Area and seven Protected Areas for Resource Management (Regional Legislative Decree No. 11/2011/A; April 20). The protected terrestrial areas cover 8,561.11 hectares, representing 21.37% of the territory (Regional-Directorate-for-the-Environment 2020). In 2011, Matela Forest, located on an ancient lava flow, was designated as a Protected Area for Habitat or Species Management given that its natural values, high biodiversity indices and representation in terms of flora (Regional Legislative Decree No. 11/2011/A; April 20). The vegetation of Matela can be classified as sub-montane forest, usually dominated by *Laurusazorica* (Azorean Laurel forests) that, in the past, probably covered more than two-thirds of the Azorean islands, from 300 m to 600 a.s.l. (Elias et al. 2016). For example, Matela Forest represents a "hotspot" of species, exhibiting a high specific richness for bryophytes, including globally threatened species, such as the moss *Echinodiumrenauldii* (Cardot) Broth. (Gabriel 1994) and could serve as a natural refuge to potentially aiding in the recolonisation of neighbouring areas (Gabriel 1994, Lloret and González-Mancebo 2011). On the leaves of laurel trees and fern fronds, it is possible to observe communities of small liverworts, including the species *Cololejeuneasintenisii* (Steph.) Pócs, equally threatened with extinction according to IUCN Criteria (González Mancebo et al. 2019). In Suppl. material 1, we list 88 species of bryophytes historically recorded for Matela.

For those visiting the area, it is impressive to observe the presence of large specimens of Azores juniper (*Juniperusbrevifolia* (Hochst. ex Seub.) Antoine subsp. brevifolia), the only endemic gymnosperm species in the Azores and one of the most important in structuring native forests. A preliminary dendrochronological analysis (Elias, unpublished data) suggests that these trees are likely over 150 years old (see Suppl. material 2 for the listing of the 42 species of vascular plants already referenced for Matela).

Based on historical data (2002 sampling - see Pozsgai et al. (2024) - and other sources), it is worth highlighting the presence of a significant number of endemic arthropod species (about 23 species; Suppl. material 3), including eight species of spiders, which represent 60% of the known endemic species on Terceira Island (*Canariphantesacoreensis* (Wunderlich, 1992); *Savigniorrhipisacoreensis* Wunderlich, 1992; *Emblynaacoreensis* Wunderlich, 1992; *Gibbaraneaoccidentalis* Wunderlich, 1989; *Lasaeolaoceanica* Simon, 1883; *Leucognathaacoreensis* Wunderlich, 1992; *Pardosaacorensis* Simon, 1883; *Rugathodesacoreensis* Wunderlich, 1992) with the first three being classified as vulnerable by IUCN Criteria. Amongst the beetles (Insecta, Coleoptera), four relatively rare endemic species mentioned in literature stand out, all classified as endangered by IUCN, namely: *Athousazoricus* Platia & Gudenzi, 2002; *Atlantocisgillerforsi* Israelson, 1986; *Drouetiusborgesiborgesi* Machado, 2009; and *Pseudechinosomanodosum* Hustache, 1936.

Amongst mammals, the endemic bat *Nyctalusazoreum* (Thomas, 1901) can be observed in Matela, a diurnal insectivorous species considered Vulnerable by the IUCN. Nine species of native birds are reported for the area, including the endemic subspecies of goldcrest, *Regulusregulusinermis* (Murphy & Chapin, 1929) and the Azores chaffinch, *Fringillacoelebsmoreletti* (Pucheran, 1859) (see Suppl. material 4).

Unfortunately, in recent years, Matela has been invaded by a high number of introduced species, possibly due to its low-altitude location, proximity to pasturelands and being a small fragment of natural vegetation with a high perimeter/area ratio, intersected by communication routes (Borges et al. 2006). These conditions may have severe consequences at the local scale, as well as for Azorean biodiversity conservation (Borges et al. 2005).

## General description

### Purpose

The main objectives of this study were: (i) provide an updated inventory of bryophytes, vascular plants, arthropods, birds and mammals within this protected area; ii) when possible, to provide some comparisons with historical data on surveys of Matela.

## Project description

### Title

Multitaxa Inventory of Matela (Terceira, Azores, Portugal) - Protected Area for the Management of Habitats or Species.

### Personnel

Fieldwork (site selection and experimental setting): Rosalina Gabriel (Bryophytes); Rui B. Elias (Vascular Plants); Paulo A. V. Borges (Arthropods) & Mariana Sousa (Vertebrates).

Fieldwork (authorisation): Azorean Minister of Environment (Lic 46/2022/DRAAC) and Azorean Minister of Science and Technology (CCPI 28/2022/DRCT).

Fieldwork (sample collection): Bryophytes (Mariana Sousa, Bruna Martins & Rosalina Gabriel); Vascular Plants (Mariana Sousa, Joana Romão, Joana Roxo, Bruna Martins & Rui B. Elias); Arthropods (Mariana Sousa & Paulo A.V. Borges); Vertebrates (Mariana Sousa, Bruna Martins & Lucas Lamelas-López).

Parataxonomists: Bryophytes (Mariana Sousa); Vascular Plants (Joana Romão, Joana Roxo & Mariana Sousa); Arthropods (Mariana Sousa & Abrão Leite); Vertebrates (Bruna Martins & Mariana Sousa).

Taxonomist: Rosalina Gabriel (Bryophytes); Rui B. Elias (Vascular Plants); Paulo A. V. Borges (Arthropods) & Mariana Sousa (Vertebrates).

Voucher specimen management: Bryophytes (Mariana Sousa & Rosalina Gabriel); Arthropods (Mariana Sousa & Abrão Leite).

Database management: Mariana Sousa, Rosalina Gabriel, Paulo A. V. Borges & Rui B. Elias.

Darwin Core databases management: Lucas Lamelas-López, Rosalina Gabriel & Paulo A. V. Borges.

### Study area description

Matela (Latitude: 38°41'59"N, Longitude: 27°15'40"W) is located at low-medium altitude (300-400 m a.s.l.) inland of Terceira Island (Fig. 1), the third largest island of the Azorean Archipelago (about 400 km^2^). Matela is included in the Natural Park of Terceira Island and is considered a Protected Area for the Management of Habitats or Species (Regional Legislative Decree nº 11/2011/A, of 20 April 2011). It has a total area of 220,530 m^2^ and, although the main habitat comprises natural forests, mainly dominated by *Ericaazorica* Hochst. ex Seub. or *Laurusazorica* (Seub.) Franco, it also includes small patches of *Eucalyptusglobulus* Labill. and Japanese cedar (*Cryptomeriajaponica* D.Don) plantations and semi-natural pastures (Elias et al. 2016).

### Design description

The sampling protocol was carried out in 2022 and it is based on GIMS - A Global Island Monitoring Scheme protocols (Borges et al. 2018) (see more details below).

The historical data were obtained from herbarium and other unpublished data and through a bibliographic revision (from 1970 to date) (see list of main sources in Suppl. material 5). Particularly for the bryophytes, data come from the study of Gabriel and Bates (2005). More detailed data from arthropods sampled in 2020 can be consulted in Pozsgai et al. (2024).

### Funding

FCT-UIDB/00329/2020-2024 (https://doi.org/10.54499/UIDB/00329/2020); Azores DRCT Pluriannual Funding (M1.1.A/FUNC.UI&D/010/2021-2024); Viridia – Conservation In Action Contract “Matela – uma ilha de biodiversidade”.

## Sampling methods

### Study extent

Most sampling was performed in 2022 on the “Protected Area for the Management of Habitats or Species of Matela”, located on Terceira Island (Azores, Portugal). This area is the unique available small fragment of native forest located at a low-medium altitude (300-400 m a.s.l.). Unfortunately, this small fragment was recently invaded by exotic invasive plant species.

### Sampling description

The sampling protocol is based on GIMS - A Global Island Monitoring Scheme protocols (Borges et al. 2018).

For bryophytes, in the summer of 2022, three quadrats of 2 m × 2 m were sampled in three habitats: native forest, grassland and a former eucalyptus plantation; in each quadrat, three samples (microplots) of 10 cm × 5 cm were collected per substrate type, tottaling 71 samples with bryophytes. For vascular plants, inventories were made in 72 sub-plots measuring 5 m × 5 m, in addition to a list of all observed species. Sampling mostly occurred in the autumn of 2022, but some data from a previous vascular plant survey in spring 2015 were also included.

**Figure 2.**
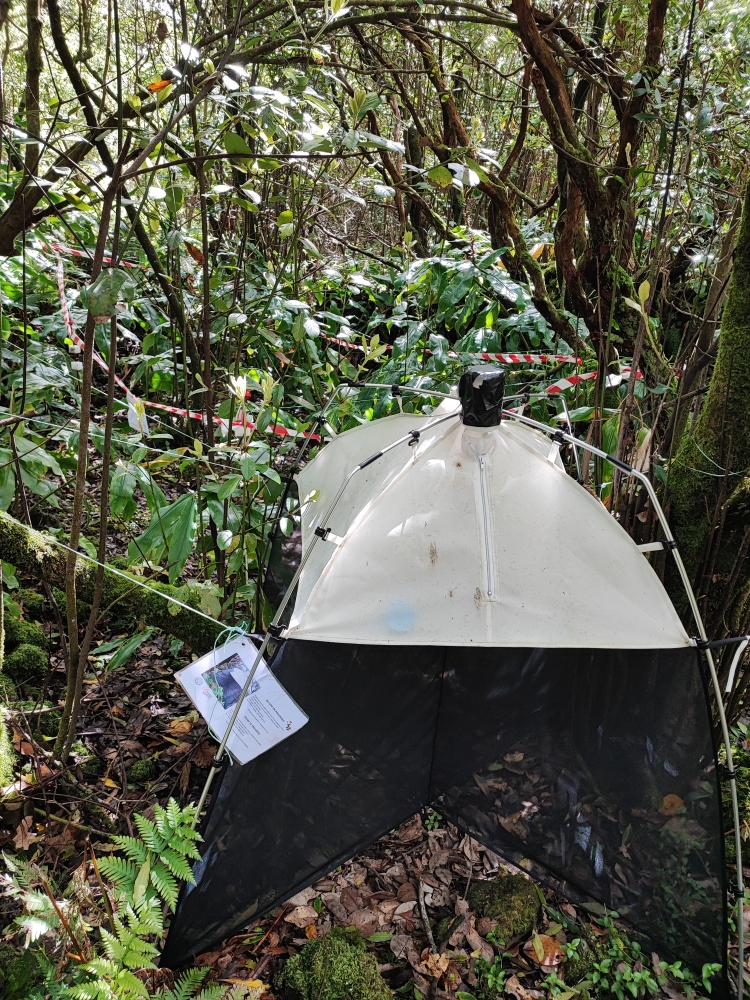
SLAM trap (Sea, Land, Air, Malaise trap) (Credit: Paulo A. V. Borges).

For sampling arthropods, in the summer of 2022, the BALA methodology was used (Borges et al. 2005, Borges et al. 2018): 30 pitfall traps were set to sample soil arthropods and the canopy of the dominant tree species (*Juniperusbervifolia*, *Ericaazorica*, *Laurusazorica*, *Pittosporumundulatum*) was beaten to collect plant-associated arthropods (10 samples per tree species); in addition to this protocol, one flight interception trap (SLAM - Sea, Land, Air, Malaise traps) (Fig. 2) was also used to sample flying arthropods or arthropods with great dispersal capacity. This SLAM trap has been operating since 2019 (see Borges et al. (2022a)), but the sample used for the current study was the sample from the summer of 2022.

The bird census was carried out at 25 observation points, always between 07:00 and 11:00 am during the summer and autumn 2022, applying the listening point method and other observations. The mammal inventory was carried out using camera traps at 30 sampling points also during the summer and autumn 2022. All the details of the sites can be consulted in the event table in GBIF (Sousa et al. 2024).

### Quality control

All collected specimens were identified or revised by a taxonomical expert.

### Step description

For Bryophytes, the final validation was made by Rosalina Gabriel. For Vascular Plants, the final validation was made by Rui B. Elias. For arthropods, the final validation was made by Paulo A. V. Borges. For Birds and Mammals, the identification was made by Mariana Sousa.

The nomenclature and colonisation status of species follows the most updated information available in the AZORESBIOPORTAL (https://azoresbioportal.uac.pt/). For arthropods, this information is also available in the last published checklist (Borges et al. 2022b).

## Geographic coverage

### Description

The study was conducted on the Protected Area for the Management of Habitats or Species of Matela, Terceira Island, Azores, (Portugal).

### Coordinates

38.700538 and 38.69533 Latitude; -27.26478 and -2725338 Longitude.

## Taxonomic coverage

### Description

We have covered several taxonomic groups, namely mosses and liverworts (Bryophyta, Marchantiophyta), vascular plants (Magnoliophyta, Lycopodiophyta, Pteridophyta, Pinophyta), arthropods (Arthropoda) and vertebrates (Chordata).

### Taxa included

**Table:** 

Rank	Scientific Name	Common Name
phylum	Bryophyta	Mosses
phylum	Marchantiophyta	Liverworts
phylum	Lycopodiophyta	Ferns and alies
phylum	Pteridophyta	Ferns
phylum	Pinophyta	Conifers
phylum	Magnoliophyta	Flowering plants
phylum	Arthropoda	Arthropods
phylum	Chordata	Birds and mammals

## Temporal coverage

### Notes

01-07-2015 - 15-11-2022

## Collection data

### Collection name

AZU_Section Bryophytes; AZU_Section Vascular Plants; Dalberto Teixeira Pombo (Arthropods)

### Collection identifier

AZU (Bryophytes and Vascular Plants); DTP (Arthropods)

### Specimen preservation method

Dry (Bryophytes and Vascular Plants); Ethanol 96% (Arthropods).

### Curatorial unit

Curator: Rosalina Gabriel (Bryophytes); Rui B. Elias (Vascular Plants); Paulo A. V. Borges (Arthropods).

## Usage licence

### Usage licence

Creative Commons Public Domain Waiver (CC-Zero)

## Data resources

### Data package title

Biodiversity inventory of the Protected Area for the Management of Habitats or Species of Matela (Terceira, Azores, Portugal)

### Resource link

https://doi.org/10.15468/qbj3rd

### Alternative identifiers

http://ipt.gbif.pt/ipt/resource?r=matela_project; https://www.gbif.org/dataset/30ff08cc-4913-4564-a84c-734b040b9380

### Number of data sets

2

### Data set 1.

#### Data set name

Event Table

#### Data format

Darwin Core Archive format

#### Character set

UTF-8

#### Download URL

http://ipt.gbif.pt/ipt/resource?r=matela_project

#### Data format version

1.5

#### Description

The dataset was published in the Global Biodiversity Information Facility platform, GBIF (Sousa et al. 2024). The following data table includes all the records for which a taxonomic identification of the species was possible. The dataset submitted to GBIF is structured as a sample event dataset that has been published as a Darwin Core Archive (DwCA), which is a standardised format for sharing biodiversity data as a set of one or more data tables. The core data file contains 269 records (eventID). This GBIF IPT (Integrated Publishing Toolkit, Version 2.5.6) archives the data and, thus, serves as the data repository. The data and resource metadata are available for download in the Portuguese GBIF Portal IPT (Sousa et al. 2024).

**Data set 1.** 

Column label	Column description
eventID	Identifier of the events, unique for the dataset.
locationID	Identifier of the location.
stateProvince	Name of the region of the sampling site (Azores).
islandGroup	Name of archipelago (Azores).
island	Name of the island (Terceira).
country	Country of the sampling site (Portugal).
countryCode	ISO code of the country of the sampling site (PT).
municipality	Municipality of the sampling sites (Angra do Heroísmo).
minimumElevationInMetres	The lower limit of the range of elevation (altitude, usually above sea level), in metres.
decimalLongitude	Approximate centre point decimal longitude of the field site in GPS coordinates.
decimalLatitude	Approximate centre point decimal latitude of the field site in GPS coordinates.
geodeticDatum	The ellipsoid, geodetic datum or spatial reference system (SRS), upon which the geographic coordinates given in decimalLatitude and decimalLongitude are based.
coordinateUncertaintyInMetres	Uncertainty of the coordinates of the centre of the sampling plot.
coordinatePrecision	Precision of the coordinates.
georeferenceSources	A list (concatenated and separated) of maps, gazetteers or other resources used to georeference the Location, described specifically enough to allow anyone in the future to use the same resources.
locality	Name of the locality.
habitat	The habitat of the sample.
day	Day of the event.
month	Month of the event.
year	Year of the event.
eventDate	Date or date range the record was collected.
sampleSizeValue	The numeric amount of time spent in each sampling.
sampleSizeUnit	The unit of the sample size value.
verbatimEventDate	The verbatim original representation of the date and time information for an Event. In this case, we use the season and year.
samplingProtocol	The sampling protocol used to capture the species.

### Data set 2.

#### Data set name

Occurrence Table

#### Data format

Darwin Core Archive format

#### Character set

UTF-8

#### Download URL

http://ipt.gbif.pt/ipt/resource?r=matela_project

#### Data format version

1.5

#### Description

The dataset was published in the Global Biodiversity Information Facility platform, GBIF (Sousa et al. 2024). The following data table includes all the records for which a taxonomic identification of the species was possible. The dataset submitted to GBIF is structured as an occurrence table that has been published as a Darwin Core Archive (DwCA), which is a standardised format for sharing biodiversity data as a set of one or more data tables. The core data file contains 1801 records (occurrenceID). This GBIF IPT (Integrated Publishing Toolkit, Version 2.5.6) archives the data and, thus, serves as the data repository. The data and resource metadata are available for download in the Portuguese GBIF Portal IPT (Sousa et al. 2024).

**Data set 2.** 

Column label	Column description
eventID	Identifier of the events, unique for the dataset.
type	Type of the record, as defined by the Public Core standard.
licence	Reference to the licence under which the record is published.
institutionID	The identity of the institution publishing the data.
collectionID	The identity of the collection publishing the data.
institutionCode	The code of the institution publishing the data.
collectionCode	The code of the collection where the specimens are conserved.
basisOfRecord	The nature of the data record.
occurrenceID	Identifier of the record, coded as a global unique identifier.
organismQuantity	A number or enumeration value for the quantity of organisms.
organismQuantityType	The type of quantification system used for the quantity of organisms. For bryophytes, we used the Braun Blanquet Scale.
sex	The sex and quantity of the individuals captured.
lifeStage	The life stage of the organisms captured.
establishmentMeans	The process of establishment of the species in the location, using a controlled vocabulary: 'native', 'introduced', 'endemic', "indeterminate".
dynamicProperties	Additional information about the process of the establishment of the species.
recordedBy	A list (concatenated and separated) of names of people, groups or organisations who performed the sampling in the field.
identifiedBy	A list (concatenated and separated) of names of people, groups or organisations who assigned the Taxon to the subject.
dateIdentified	The date on which the subject was determined as representing the Taxon.
kingdom	Kingdom name.
phylum	Phylum name.
class	Class name.
order	Order name.
family	Family name.
genus	Genus name.
scientificName	Species name.
specificEpithet	Specific epithet.
infraspecificEpithet	Infraspecific epithet.
scientificNameAuthorship	Name of the author of the lowest taxon rank included in the record.
taxonRank	Lowest taxonomic rank of the record.
identificationRemarks	Information about arthropod morphospecies identification (code in Dalberto Teixeira Pombo Collection).

## Additional information

**Results**

Overall two kindgoms, six phyla, 14 classes, 58 orders and 254 taxa are listed. A total of 46 species are endemic distributed as follows: bryophytes - 4; vascular plants - 15; arthropods - 17; vertebrates - 10.

In the community of bryophytes, we recorded a total of 75 taxa, comprising 43 mosses and 32 liverworts. Amongst these, 71 were indigenous, while three remained indeterminate and one, *Campylopusintroflexus*, was identified as invasive (Table 1). The most frenquent species in the plots were the liverworts *Frullaniaacicularis* Hentschel & von Konrat (n = 26) and *Heteroscyphusdenticulatus* (Mitt.) Schiffn. (n = 26). A temporal comparison with previous data (Suppl. material 1) unveiled a decrease in richness, offset by the addition of 23 new species to the area.

Concerning vascular plants, we identified 54 species, with 28 being indigenous and 26 introduced (Table 2). The most frequent species in plots were the endemic fern *Dryopterisazorica* (Christ) Alston (n = 64) and the endemic tree *Laurusazorica* (Seub.) Franco (n = 63). However, several exotic invasive species are also very frequent, namely *Pittosporumundulatum* Vent. (n = 54), *Rubusulmifolius* Schott (n = 49) and *Hedychiumgardnerianum* Sheppard ex Ker-Gawl. (n = 43). Of the 54, a remarkable 43 were newly recorded for Matela (see historical records in Suppl. material 2).

The study of arthropods encompassed a total of 103 taxa (Table 3). The most abundant species were the introduced *Stelidotageminata* (Say, 1825) (Insecta, Coleoptera; n = 935) and the native *Lasiusgrandis* Forel, 1909 (Insecta, Hymenoptera; n = 263). Within the community of soil arthropods, we documented eight indigenous and 25 introduced taxa, witnessing the disappearance of endemic species alongside a substantial increase in introduced ones between 2002 and 2022 (see Suppl. material 3). Canopy arthropods, totalling 36 indigenous and 18 introduced taxa, exhibited a similar trend in the increase of introduced species but not in the loss of endemic species. SLAM traps captured 24 indigenous and 15 introduced arthropod taxa.

In relation to Chordata, avian species totalled 12 indigenous and one introduced bird, indicating an inrease in the overall taxa count for the area (Table 4 and Suppl. material 4). The most abundant bird species were the endemic Passeriformes *Turdusmerulaazorensis* Hartert, E, 1905 (n = 134) and *Fringillacoelebsmoreletti* Pucheran, 1859 (n = 128). Finally, the mammalian species included eight introduced species, marking new records for Matela, the most abundant being *Rattusrattus* (Linnaeus, 1758) (n = 223) and one endemic species, the Azorean endemic bat *Nyctalusazoreum* (n = 3).

Despite Matela harbouring numerous native and endemic species, thereby maintaining a highly notable natural heritage, it has recently fallen object to the invasion of introduced exotic species, some of them also invasive. Of high concern is the spread of *Pittosporumundulatum* Vent. (n = 54), *Rubusulmifolius* Schott (n = 49) and *Hedychiumgardnerianum* Sheppard ex Ker-Gawl.

The number of recorded species has substantially increased across almost all groups for which historical data allow comparisons. However, we were not able to confirm the presence of some epigean endemic arthropods that were sampled in 2002, namely three spider species (*Canariphantesacoreensis* (Wunderlich, 1992); *Lasaeolaoceanica* Simon, 1883; *Pardosaacorensis* Simon, 1883) and several beetle species, namely *Athousazoricus* Platia & Gudenzi, 2002; *Atlantocisgillerforsi* Israelson, 1986; *Drouetiusborgesiborgesi* Machado, 2009; and *Pseudechinosomanodosum* Hustache, 1936.

The genuine impact of the exotic potentially invasive species on native communities remains not fully clarified; nevertheless, the imperative to address this pressing issue for nature conservation in the Azores is unequivocal. Within the project “Matela – uma ilha de biodiversidade” (“Matela - an island of biodiversity”), we aim to contribute to the restoration of this important fragment of native forest and we will maintain the monitoring of the several taxonomic groups during the next years using the same protocols.

## Supplementary Material

21DCF1CD-9AF7-5016-B0E2-B76222CA69E8 10.3897/BDJ.12.e121884.suppl1 Supplementary material 1 List of bryophytes historically documented in Matela (Bryophyta, Marchantiophyta and Anthocerotophyta) Data type Occurrences Brief description Detailed list of bryophytes found in Matela, based on a revision of historical literature, including grey literature. File: oo_1005698.docx https://binary.pensoft.net/file/1005698 Mariana Sousa & Rosalina Gabriel

F8ECC7DD-885F-554F-9752-2BC551A8BD96 10.3897/BDJ.12.e121884.suppl2 Supplementary material 2 List of Vascular Plants historically recorded in Matela (Lycopodiophyta, Pteridophyta, Pinophyta and Magnoliophyta) Data type Occurrences Brief description Detailed list of vascular plants found in Matela, based on a revision of historical literature, including grey literature. File: oo_1005700.docx https://binary.pensoft.net/file/1005700 Mariana Sousa & Rui Bento Elias

A8AB21FE-379C-56C3-BBA1-AB1725CA02B7 10.3897/BDJ.12.e121884.suppl3 Supplementary material 3 List of arthropods historically documented in Matela (Arthropoda) Data type Occurrences Brief description Detailed list of arthropods found in Matela, based on a revision of historical literature, including grey literature. File: oo_1005706.docx https://binary.pensoft.net/file/1005706 Mariana Sousa & Paulo A.V. Borges

49D2CA82-FB5C-527E-9F93-04F88581742E 10.3897/BDJ.12.e121884.suppl4 Supplementary material 4 List of vertebrates historically documented in Matela Data type Occurrence Brief description Detailed list of vertebrate found in Matela, based on a revision of historical literature, including grey literature. File: oo_1005707.docx https://binary.pensoft.net/file/1005707 Mariana Sousa

72C92905-9ACB-5E87-B654-7C2FC2B5378C 10.3897/BDJ.12.e121884.suppl5 Supplementary material 5 List of historical literature sources mentioning Matela Data type Literature list Brief description List of historical literature sources mentioning Matela and that include species records. File: oo_1005711.docx https://binary.pensoft.net/file/1005711 Mariana Sousa

## Figures and Tables

**Figure 1a.**
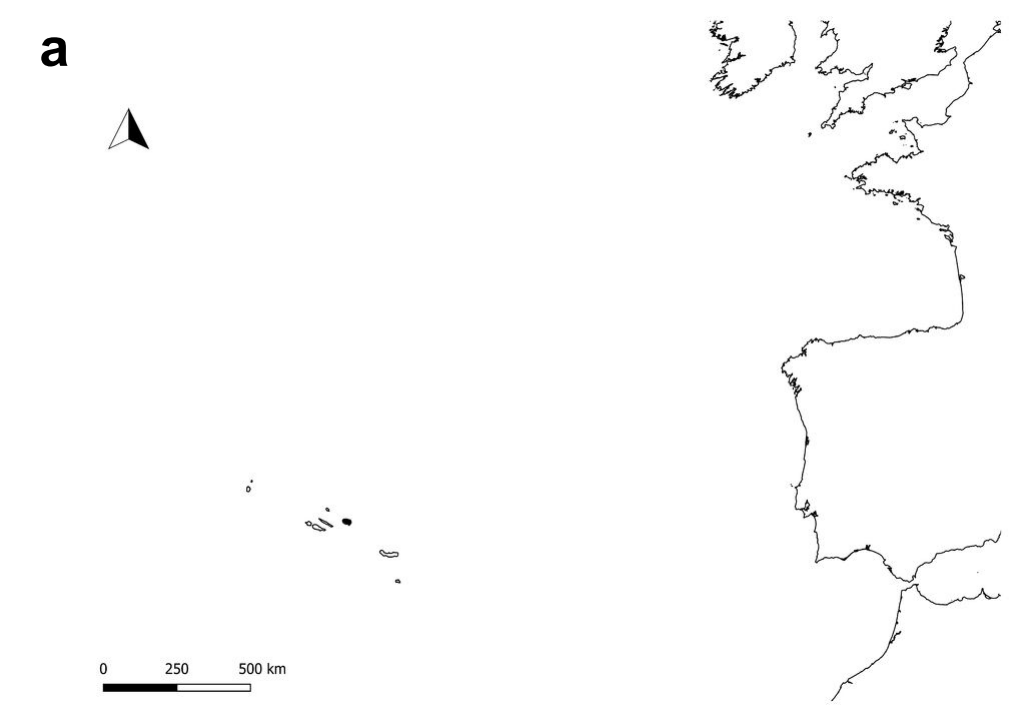
Location of Azores in North Atlantic.

**Figure 1b.**
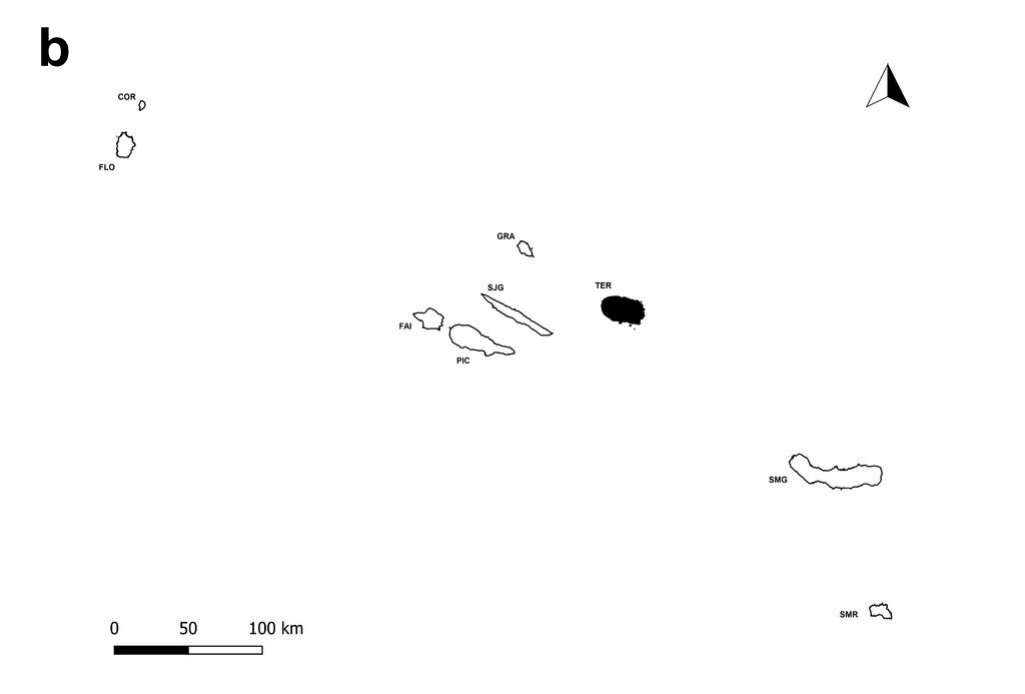
The nine Azorean Islands (COR -Corvo; FLO - Flores; FAI - Faial; PIC - Pico; SJG - São Jorge; GRA - Graciosa; TER -Terceira; SMG - São Miguel; SMR - Santa Maria). Terceira is marked in black.

**Figure 1c.**
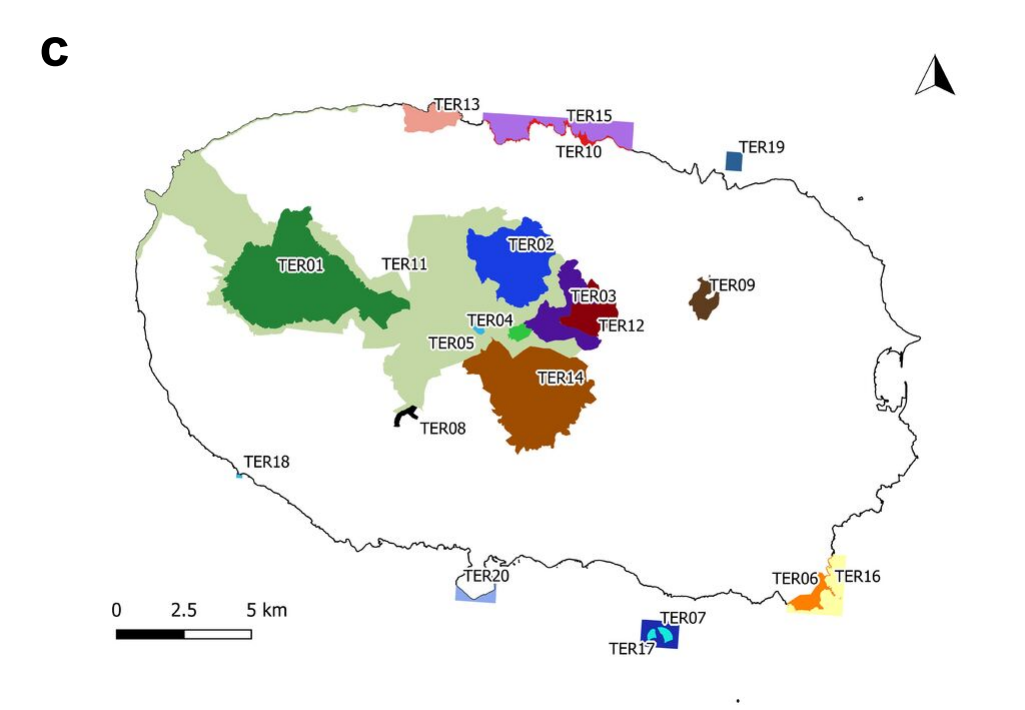
The Island of Terceira with all the protected areas and location of Matela protected area (TER08).

**Figure 1d.**
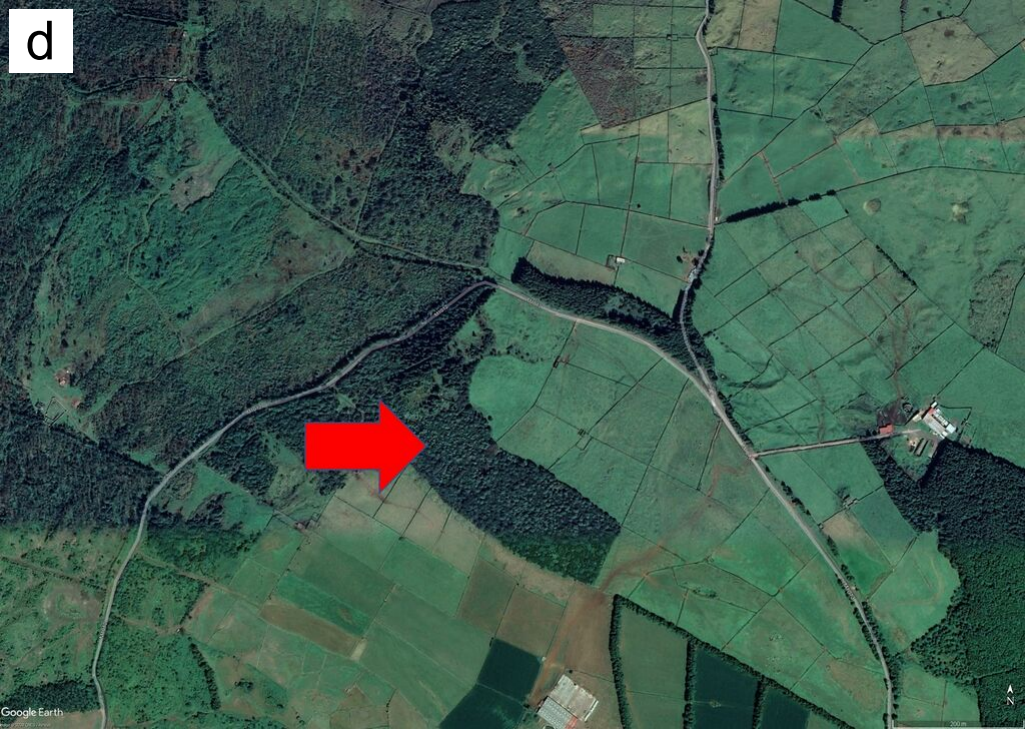
The location of Matela with an arrow locating the main sampling area.

**Table 1.** Inventory of bryophytes collected in 2022 on the Protected Area for the Management of Habitats or Species of Matela, (Natural Park of Terceira Island, Azores), including taxonomical information (Phylum, Class, Order and Species names), colonisation status (C.S.) (END - endemic from Azores; NAT - native non-endemic; INT - introduced species; IND - indeterminate origin) and number of plots where the species occurs (Plots). (Note: In the Occurrence Table, we used the Braun Blanquet Scale to determine the species abundance).

Phylum	Class	Order	Species	C.S.	Plots
Bryophyta	Bryopsida	Bryales	*Bryumruderale* Crundw. & Nyholm	NAT	1
Bryophyta	Bryopsida	Bryales	*Ptychostomumbornholmense* (Wink. & R.Ruthe) Holyoak & N.Pedersen	IND	1
Bryophyta	Bryopsida	Bryales	*Plagiomniumundulatum* (Hedw.) T.J.Kop.	NAT	1
Bryophyta	Bryopsida	Dicranales	*Dicranumflagellare* Hedw.	NAT	1
Bryophyta	Bryopsida	Dicranales	*Dicranumscottianum* Turner	NAT	1
Bryophyta	Bryopsida	Dicranales	*Ceratodonpurpureus* (Hedw.) Brid. subsp. purpureus	NAT	2
Bryophyta	Bryopsida	Dicranales	*Fissidensasplenioides* Hedw.	NAT	5
Bryophyta	Bryopsida	Dicranales	*Fissidensserrulatus* Brid.	NAT	1
Bryophyta	Bryopsida	Dicranales	*Fissidenstaxifolius* Hedw.	NAT	4
Bryophyta	Bryopsida	Dicranales	*Campylopusflexuosus* (Hedw.) Brid.	NAT	17
Bryophyta	Bryopsida	Dicranales	*Campylopusintroflexus* (Hedw.) Brid.	INT	3
Bryophyta	Bryopsida	Dicranales	*Campylopuspilifer* Brid.	NAT	1
Bryophyta	Bryopsida	Dicranales	*Campylopuspyriformis* (Schultz) Brid.	NAT	4
Bryophyta	Bryopsida	Dicranales	*Leucobryumglaucum* (Hedw.) Ångstr.	NAT	1
Bryophyta	Bryopsida	Dicranales	*Leucobryumjuniperoideum* (Brid.) Müll.Hal.	NAT	12
Bryophyta	Bryopsida	Grimmiales	*Grimmialisae* De Not.	NAT	6
Bryophyta	Bryopsida	Grimmiales	*Ptychomitriumnigrescens* (Kunze) Wijk & Margad.	NAT	1
Bryophyta	Bryopsida	Grimmiales	*Ptychomitriumpolyphyllum* (Dicks. ex Sw.) Bruch & Schimp.	NAT	3
Bryophyta	Bryopsida	Hookeriales	*Tetrastichiumvirens* (Cardot) S.P.Churchill	NAT	11
Bryophyta	Bryopsida	Hypnales	*Isotheciumprolixum* (Mitt.) M.Stech, Sim-Sim, Tangney & D.Quandt	NAT	12
Bryophyta	Bryopsida	Hypnales	*Pseudotaxiphyllumlaetevirens* (Dixon & Luisier ex F.Koppe & Düll) Hedenäs	NAT	2
Bryophyta	Bryopsida	Hypnales	*Brachytheciastrumvelutinum* (Hedw.) Ignatov & Huttunen	NAT	1
Bryophyta	Bryopsida	Hypnales	*Brachythecium* sp.	IND	1
Bryophyta	Bryopsida	Hypnales	*Brachytheciumrutabulum* (Hedw.) Schimp.	NAT	1
Bryophyta	Bryopsida	Hypnales	*Brachytheciumsalebrosum* (Hoffm. ex F.Weber & D.Mohr) Schimp.	NAT	1
Bryophyta	Bryopsida	Hypnales	*Kindbergiapraelonga* (Hedw.) Ochyra	NAT	20
Bryophyta	Bryopsida	Hypnales	*Pseudoscleropodiumpurum* (Hedw.) M.Fleisch.	NAT	5
Bryophyta	Bryopsida	Hypnales	*Rhynchostegiellaazorica* Hedenäs & Vanderp.	END	2
Bryophyta	Bryopsida	Hypnales	*Rhynchostegiumconfertum* (Dicks.) Schimp.	NAT	1
Bryophyta	Bryopsida	Hypnales	*Sciuro-hypnum populeum* (Hedw.) Ignatov & Huttunen	NAT	1
Bryophyta	Bryopsida	Hypnales	*Echinodiumrenauldii* (Cardot) Broth.	END	2
Bryophyta	Bryopsida	Hypnales	HypnumcupressiformeHedw.varcupressiforme	NAT	18
Bryophyta	Bryopsida	Hypnales	*Hypnumuncinulatum* Jur.	NAT	18
Bryophyta	Bryopsida	Hypnales	*Heterocladiumflaccidum* (Schimp.) A.J.E.Sm.	NAT	7
Bryophyta	Bryopsida	Hypnales	*Andoaberthelotiana* (Mont.) Ochyra	NAT	13
Bryophyta	Bryopsida	Hypnales	*Myuriumhochstetteri* (Schimp.) Kindb.	NAT	2
Bryophyta	Bryopsida	Hypnales	*Exsertothecaintermedia* (Brid.) S.Olsson, Enroth & D.Quandt	NAT	1
Bryophyta	Bryopsida	Hypnales	*Thamnobryumalopecurum* (Hedw.) Gangulee	NAT	1
Bryophyta	Bryopsida	Hypnales	*Thamnobryummaderense* (Kindb.) Hedenäs	NAT	1
Bryophyta	Bryopsida	Hypnales	*Thamnobryumrudolphianum* Mastracci	END	1
Bryophyta	Bryopsida	Hypnales	*Thuidiumtamariscinum* (Hedw.) Schimp.	NAT	10
Bryophyta	Polytrichopsida	Polytrichales	*Atrichumundulatum* (Hedw.) P.Beauv.	NAT	3
Bryophyta	Polytrichopsida	Polytrichales	*Polytrichumcommune* Hedw.	NAT	2
Marchantiophyta	Jungermanniopsida	Jungermanniales	*Fuscocephaloziopsiscrassifolia* (Lindenb. & Gottsche) Váňa & L.Söderstr.	NAT	1
Marchantiophyta	Jungermanniopsida	Jungermanniales	*Odontoschismasphagni* (Dicks.) Dumort.	NAT	4
Marchantiophyta	Jungermanniopsida	Jungermanniales	*Geocalyxgraveolens* (Schrad.) Nees	NAT	1
Marchantiophyta	Jungermanniopsida	Jungermanniales	*Telaraneaeuropaea* J.J.Engel & G.L.Merr.	NAT	4
Marchantiophyta	Jungermanniopsida	Jungermanniales	*Heteroscyphusdenticulatus* (Mitt.) Schiffn.	NAT	26
Marchantiophyta	Jungermanniopsida	Jungermanniales	*Lophocolea* sp.	IND	2
Marchantiophyta	Jungermanniopsida	Jungermanniales	*Lophocoleafragrans* (Moris & De Not.) Gottsche, Lindenb. & Nees	NAT	8
Marchantiophyta	Jungermanniopsida	Jungermanniales	*Lophocoleaheterophylla* (Schrad.) Dumort.	NAT	2
Marchantiophyta	Jungermanniopsida	Jungermanniales	*Plagiochilabifaria* (Sw.) Lindenb.	NAT	3
Marchantiophyta	Jungermanniopsida	Jungermanniales	*Plagiochilaexigua* (Taylor) Taylor	NAT	3
Marchantiophyta	Jungermanniopsida	Jungermanniales	*Saccogynaviticulosa* (L.) Dumort.	NAT	21
Marchantiophyta	Jungermanniopsida	Jungermanniales	*Scapaniagracilis* Lindb.	NAT	3
Marchantiophyta	Jungermanniopsida	Jungermanniales	*Scapanianemorea* (L.) Grolle	NAT	6
Marchantiophyta	Jungermanniopsida	Jungermanniopsida	*Acrobolbusazoricus* (Grolle & Perss.) Briscoe	END	3
Marchantiophyta	Jungermanniopsida	Metzgeriales	*Metzgeriafurcata* (L.) Corda	NAT	1
Marchantiophyta	Jungermanniopsida	Porellales	*Frullaniaacicularis* Hentschel & von Konrat	NAT	26
Marchantiophyta	Jungermanniopsida	Porellales	*Frullaniamicrophylla* (Gottsche) Pearson	NAT	7
Marchantiophyta	Jungermanniopsida	Porellales	*Frullaniateneriffae* (F.Weber) Nees	NAT	1
Marchantiophyta	Jungermanniopsida	Porellales	*Cololejeuneasintenisii* (Steph.) Pócs	NAT	6
Marchantiophyta	Jungermanniopsida	Porellales	*Harpalejeuneamolleri* (Steph.) Grolle	NAT	8
Marchantiophyta	Jungermanniopsida	Porellales	*Lejeuneacavifolia* (Ehrh.) Lindb.	NAT	8
Marchantiophyta	Jungermanniopsida	Porellales	*Lejeuneaeckloniana* Lindenb.	NAT	5
Marchantiophyta	Jungermanniopsida	Porellales	Lejeuneaflavasubsp.moorei (Lindb.) R.M.Schust.	NAT	1
Marchantiophyta	Jungermanniopsida	Porellales	*Lejeuneahibernica* Bischl., H.A.Mill. & Bonner ex Grolle	NAT	1
Marchantiophyta	Jungermanniopsida	Porellales	*Lejeunealamacerina* (Steph.) Schiffn.	NAT	18
Marchantiophyta	Jungermanniopsida	Porellales	*Lejeuneapatens* Lindb.	NAT	1
Marchantiophyta	Jungermanniopsida	Porellales	*Marchesiniamackaii* (Hook.) Gray	NAT	9
Marchantiophyta	Jungermanniopsida	Porellales	*Myriocoleopsisminutissima* (Sm.) R.L.Zhu, Y.Yu & Pócs	NAT	1
Marchantiophyta	Jungermanniopsida	Porellales	*Porellacanariensis* (F.Weber) Underw.	NAT	2
Marchantiophyta	Jungermanniopsida	Porellales	*Porellaobtusata* (Taylor) Trevis.	NAT	3
Marchantiophyta	Jungermanniopsida	Porellales	*Radulacarringtonii* J.B.Jack	NAT	11
Marchantiophyta	Jungermanniopsida	Porellales	*Radulawichurae* Steph.	NAT	3

**Table 2.** Inventory of vascular plants collected between 2015 and 2022, on the Protected Area for the Management of Habitats or Species of Matela, (Natural Park of Terceira Island, Azores), including taxonomical information (Kindgom, Phylum, Class, Order and Species names), colonisation status (END - endemic from Azores; NAT - native non-endemic; INT - introduced species; IND - indeterminate origin) and number of plots where the species occurs (Plots). (Note: In the Occurrence Table, we used the Braun Blanquet Scale to determine the species abundance).

Phylum	Class	Order	Species	C.E.	N
Lycopodiophyta	Selaginellopsida	Selaginellales	*Selaginellakraussiana* (Kunze) A.Braun	NAT	28
Magnoliophyta	Liliopsida	Alismatales	*Zantedeschiaaethiopica* Spreng.	INT	1
Magnoliophyta	Liliopsida	Asparagales	*Ruscusaculeatus* L.	INT	9
Magnoliophyta	Liliopsida	Liliales	*Smilaxaspera* L.	INT	1
Magnoliophyta	Liliopsida	Zingiberales	*Hedychiumgardnerianum* Sheppard ex Ker-Gawl.	INT	43
Magnoliophyta	Magnoliopsida	Apiales	*Hederaazorica* Carrière	END	6
Magnoliophyta	Magnoliopsida	Apiales	*Pittosporumundulatum* Vent.	INT	54
Magnoliophyta	Magnoliopsida	Aquifoliales	*Ilexazorica* Gand.	END	3
Magnoliophyta	Magnoliopsida	Asterales	*Erigeroncanadensis* L.	INT	3
Magnoliophyta	Magnoliopsida	Asterales	*Roldanapetasitis* (Sims) H.Rob. & Brettell	INT	1
Magnoliophyta	Magnoliopsida	Caryophyllales	*Persicariacapitata* (Buch.-Ham. Ex D.Don) H.Gross	INT	4
Magnoliophyta	Magnoliopsida	Caryophyllales	*Phytolaccaamericana* L.	INT	4
Magnoliophyta	Magnoliopsida	Ericales	*Callunavulgaris* (L.) Hull	NAT	1
Magnoliophyta	Magnoliopsida	Ericales	*Ericaazorica* Hochst. ex Seub.	END	46
Magnoliophyta	Magnoliopsida	Ericales	Lysimachiaarvensis(L.)U.Manns & Anderb.subsp.arvensis	INT	2
Magnoliophyta	Magnoliopsida	Ericales	*Lysimachiaazorica* Hornem. ex Hook.	END	30
Magnoliophyta	Magnoliopsida	Ericales	*Myrsineretusa* Aiton	END	5
Magnoliophyta	Magnoliopsida	Ericales	*Vacciniumcylindraceum* Sm.	END	1
Magnoliophyta	Magnoliopsida	Fabales	*Lotuspedunculatus* Cav.	INT	2
Magnoliophyta	Magnoliopsida	Fabales	*Morellafaya* (Aiton) Wilbur	NAT	1
Magnoliophyta	Magnoliopsida	Fabales	*Quercusrobur* L.	INT	1
Magnoliophyta	Magnoliopsida	Fabales	*Trifoliumrepens* L.	INT	1
Magnoliophyta	Magnoliopsida	Gentianales	*Rubiaagostinhoi* Dansereau & P.Silva	END	2
Magnoliophyta	Magnoliopsida	Gentianales	*Vincadifformis* Pourr.	INT	2
Magnoliophyta	Magnoliopsida	Lamiales	*Digitalispurpurea* L.	INT	4
Magnoliophyta	Magnoliopsida	Lamiales	*Hallerialucida* L.	INT	12
Magnoliophyta	Magnoliopsida	Lamiales	*Marrubiumvulgare* L.	INT	2
Magnoliophyta	Magnoliopsida	Lamiales	*Menthasuaveolens* Ehrh.	INT	5
Magnoliophyta	Magnoliopsida	Lamiales	*Picconiaazorica* (Tutin) Knobl.	END	9
Magnoliophyta	Magnoliopsida	Lamiales	*Plantagolanceolata* L.	INT	2
Magnoliophyta	Magnoliopsida	Laurales	*Laurusazorica* (Seub.) Franco	END	63
Magnoliophyta	Magnoliopsida	Myrtales	*Eucalyptusglobulus* Labill.	INT	4
Magnoliophyta	Magnoliopsida	Myrtales	*Psidiumcattleyanum* Sabine	INT	1
Magnoliophyta	Magnoliopsida	Rosales	*Fragariavesca* L.	NAT	3
Magnoliophyta	Magnoliopsida	Rosales	*Frangulaazorica* Grubov	END	4
Magnoliophyta	Magnoliopsida	Rosales	*Rubusulmifolius* Schott	INT	49
Magnoliophyta	Magnoliopsida	Saxifragales	*Umbilicus rupestris* (Salisb.) Dandy	NAT	4
Magnoliophyta	Magnoliopsida	Solanales	*Solanummauritianum* Scop.	INT	2
Pinophyta	Pinopsida	Pinales	*Cryptomeriajaponica* D.Don	INT	6
Pinophyta	Pinopsida	Pinales	Juniperusbrevifolia(Hochst. ex Seub.)Antoinesubsp.brevifolia	END	2
Pteridophyta	Polypodiopsida	Cyatheales	*Sphaeropteriscooperi* (F. Muell.) R.M.Tryon	INT	4
Pteridophyta	Polypodiopsida	Hymenophyllales	*Hymenophyllumtunbrigense* (L.) Sm.	NAT	11
Pteridophyta	Polypodiopsida	Hymenophyllales	*Vandenboschiaspeciosa* (Willd.) G.Kunkel	NAT	1
Pteridophyta	Polypodiopsida	Polypodiales	*Aspleniumazoricum* (Milde) Lovis, Rasbach & Reichst.	END	7
Pteridophyta	Polypodiopsida	Polypodiales	*Aspleniumscolopendrium* L.	NAT	25
Pteridophyta	Polypodiopsida	Polypodiales	*Doodiacaudata* (Cav.) R.Br.	INT	2
Pteridophyta	Polypodiopsida	Polypodiales	*Dryopterisaemula* (Aiton) Kuntze	NAT	14
Pteridophyta	Polypodiopsida	Polypodiales	*Dryopterisaffinis* (Lowe) Fraser-Jenk.	NAT	10
Pteridophyta	Polypodiopsida	Polypodiales	*Dryopterisazorica* (Christ) Alston	END	64
Pteridophyta	Polypodiopsida	Polypodiales	*Dryopteriscrispifolia* Rasbach, Reichst. & Vida	END	2
Pteridophyta	Polypodiopsida	Polypodiales	Polypodiummacaronesicumsubsp.azoricum (Vasc.) Rumsey, Carine & Robba	END	16
Pteridophyta	Polypodiopsida	Polypodiales	*Pteridiumaquilinum* (L.) Kuhn	NAT	23
Pteridophyta	Polypodiopsida	Polypodiales	*Pterisincompleta* Cav.	NAT	11
Pteridophyta	Polypodiopsida	Polypodiales	*Struthiopterisspicant* (L.) Weis	NAT	6

**Table 3.** Arthropod inventory collected in 2022, on the Protected Area for the Management of Habitats or Species of Matela, (Natural Park of Terceira Island, Azores), including taxonomical information (Kindgom, Phylum, Class, Order and Species names), colonisation status (C.S.) (END - endemic from Azores; NAT - native non-endemic; INT - introduced species; IND - indeterminate origin) and overall abundance data (N) (Note: only taxa identified at species level are included).

Phylum	Class	Order	Species	C.S.	N
Arthropoda	Arachnida	Araneae	*Cheiracanthiumerraticum* (Walckenaer, 1802)	INT	58
Arthropoda	Arachnida	Araneae	*Clubionaterrestris* Westring, 1851	INT	4
Arthropoda	Arachnida	Araneae	*Cryptachaeablattea* (Urquhart, 1886)	INT	2
Arthropoda	Arachnida	Araneae	*Dysderacrocata* C.L.Koch, 1838	INT	1
Arthropoda	Arachnida	Araneae	*Emblynaacoreensis* Wunderlich, 1992	END	65
Arthropoda	Arachnida	Araneae	*Entelecaraschmitzi* Kulczynski, 1905	NAT	6
Arthropoda	Arachnida	Araneae	*Erigoneatra* Blackwall, 1833	INT	1
Arthropoda	Arachnida	Araneae	*Gibbaraneaoccidentalis* Wunderlich, 1989	END	18
Arthropoda	Arachnida	Araneae	*Lathysdentichelis* (Simon, 1883)	NAT	177
Arthropoda	Arachnida	Araneae	*Leucognathaacoreensis* Wunderlich, 1992	END	1
Arthropoda	Arachnida	Araneae	*Macaroeriscata* (Blackwall, 1867)	NAT	32
Arthropoda	Arachnida	Araneae	*Macaroerisdiligens* (Blackwall, 1867)	NAT	13
Arthropoda	Arachnida	Araneae	*Mangoraacalypha* (Walckenaer, 1802)	INT	2
Arthropoda	Arachnida	Araneae	*Metellinamerianae* (Scopoli, 1763)	INT	1
Arthropoda	Arachnida	Araneae	*Nigmapuella* (Simon, 1870)	INT	2
Arthropoda	Arachnida	Araneae	*Palliduphantesschmitzi* (Kulczynski, 1899)	NAT	50
Arthropoda	Arachnida	Araneae	*Pelecopsisparallela* (Wider, 1834)	INT	18
Arthropoda	Arachnida	Araneae	*Porrhoclubionadecora* (Blackwall, 1859)	NAT	61
Arthropoda	Arachnida	Araneae	*Rugathodesacoreensis* Wunderlich, 1992	END	3
Arthropoda	Arachnida	Araneae	*Savigniorrhipisacoreensis* Wunderlich, 1992	END	17
Arthropoda	Arachnida	Araneae	*Steatodanobilis* (Thorell, 1875)	NAT	1
Arthropoda	Arachnida	Araneae	*Tenuiphantesmiguelensis* (Wunderlich, 1992)	NAT	5
Arthropoda	Arachnida	Araneae	*Tenuiphantestenuis* (Blackwall, 1852)	INT	29
Arthropoda	Arachnida	Araneae	*Theridionmusivivum* Schmidt, 1956	NAT	1
Arthropoda	Arachnida	Araneae	*Xysticuscor* Canestrini, 1873	NAT	1
Arthropoda	Arachnida	Araneae	*Zygiellax-notata* (Clerck, 1757)	INT	2
Arthropoda	Arachnida	Opiliones	*Homalenotuscoriaceus* (Simon, 1879)	NAT	1
Arthropoda	Arachnida	Opiliones	*Leiobunumblackwalli* Meade, 1861	NAT	41
Arthropoda	Arachnida	Pseudoscorpiones	*Chthoniusischnocheles* (Hermann, 1804)	INT	1
Arthropoda	Arachnida	Pseudoscorpiones	*Ephippiochthoniustetrachelatus* (Preyssler, 1790)	INT	2
Arthropoda	Chilopoda	Lithobiomorpha	*Lithobiuspilicornispilicornis* Newport, 1844	NAT	1
Arthropoda	Diplopoda	Julida	*Blaniulusguttulatus* (Fabricius, 1798)	INT	161
Arthropoda	Diplopoda	Julida	*Brachyiuluspusillus* (Leach, 1814)	INT	2
Arthropoda	Diplopoda	Julida	Cylindroiulus propinquus (Porat, 1870)	INT	15
Arthropoda	Diplopoda	Julida	Nopoiulus kochii (Gervais, 1847)	INT	79
Arthropoda	Diplopoda	Julida	*Ommatoiulusmoreleti* (Lucas, 1860)	INT	7
Arthropoda	Diplopoda	Polydesmida	*Oxidusgracilis* (C.L. Koch, 1847)	INT	260
Arthropoda	Diplopoda	Polydesmida	*Polydesmuscoriaceus* Porat, 1870	INT	94
Arthropoda	Insecta	Archaeognatha	*Diltasaxicola* (Womersley, 1930)	NAT	4
Arthropoda	Insecta	Blattodea	*Zethasimonyi* (Krauss, 1892)	NAT	71
Arthropoda	Insecta	Coleoptera	*Aleocharabipustulata* (Linnaeus, 1760)	IND	2
Arthropoda	Insecta	Coleoptera	*Anaspisproteus* Wollaston, 1854	NAT	1
Arthropoda	Insecta	Coleoptera	*Anotylusnitidifrons* (Wollaston, 1871)	IND	11
Arthropoda	Insecta	Coleoptera	*Athetaaeneicollis* (Sharp, 1869)	IND	1
Arthropoda	Insecta	Coleoptera	*Brachypeplusmauli* Gardner & Classey, 1962	INT	2
Arthropoda	Insecta	Coleoptera	*Calacallessubcarinatus* (Israelson, 1984)	END	3
Arthropoda	Insecta	Coleoptera	*Catopscoracinus* Kellner, 1846	NAT	4
Arthropoda	Insecta	Coleoptera	*Cercyonhaemorrhoidalis* (Fabricius, 1775)	INT	2
Arthropoda	Insecta	Coleoptera	*Coccotrypescarpophagus* (Hornung, 1842)	INT	1
Arthropoda	Insecta	Coleoptera	*Cryptamorphadesjardinsii* (Guérin-Méneville, 1844)	INT	2
Arthropoda	Insecta	Coleoptera	*Dryopsalgiricus* (Lucas, 1846)	NAT	1
Arthropoda	Insecta	Coleoptera	*Epitrixhirtipennis* (Melsheimer, 1847)	INT	1
Arthropoda	Insecta	Coleoptera	*Epuraeabiguttata* (Thunberg, 1784)	INT	4
Arthropoda	Insecta	Coleoptera	*Longitarsuskutscherai* (Rye, 1872)	INT	1
Arthropoda	Insecta	Coleoptera	*Ocypusaethiops* (Waltl, 1835)	IND	1
Arthropoda	Insecta	Coleoptera	*Ocysharpaloides* (Audinet-Serville, 1821)	NAT	2
Arthropoda	Insecta	Coleoptera	*Phenolialimbatatibialis* (Boheman, 1851)	INT	5
Arthropoda	Insecta	Coleoptera	*Phloeonomuspunctipennis* C.G.Thomson, 1867	IND	2
Arthropoda	Insecta	Coleoptera	*Popilliajaponica* Newman, 1838	INT	1
Arthropoda	Insecta	Coleoptera	*Pseudophloeophagustenax*borgesi Stüben, 2022	END	31
Arthropoda	Insecta	Coleoptera	*Rugilusorbiculatus* (Paykull, 1789)	IND	1
Arthropoda	Insecta	Coleoptera	*Sericoderuslateralis* (Gyllenhal, 1827)	INT	1
Arthropoda	Insecta	Coleoptera	*Stelidotageminata* (Say, 1825)	INT	935
Arthropoda	Insecta	Coleoptera	*Tachyporusnitidulus* (Fabricius, 1781)	IND	2
Arthropoda	Insecta	Dermaptera	*Forficulaauricularia* Linnaeus, 1758	INT	3
Arthropoda	Insecta	Hemiptera	*Campyloneuravirgula* (Herrich-Schaeffer, 1835)	NAT	1
Arthropoda	Insecta	Hemiptera	*Cinarajuniperi* (De Geer, 1773)	NAT	7
Arthropoda	Insecta	Hemiptera	*Cixiusazoterceirae* Remane & Asche, 1979	END	23
Arthropoda	Insecta	Hemiptera	*Cyphopterumadcendens* (Herrich-Schäffer, 1835)	NAT	2
Arthropoda	Insecta	Hemiptera	*Kleidocerysericae* (Horváth, 1909)	NAT	9
Arthropoda	Insecta	Hemiptera	*Megamelodesquadrimaculatus* (Signoret, 1865)	NAT	1
Arthropoda	Insecta	Hemiptera	*Piezodoruslituratus* (Fabricius, 1794)	NAT	6
Arthropoda	Insecta	Hemiptera	*Pinalitusoromii* J. Ribes, 1992	END	1
Arthropoda	Insecta	Hemiptera	*Saldulapalustris* (Douglas, 1874)	NAT	1
Arthropoda	Insecta	Hemiptera	*Siphantaacuta* (Walker, 1851)	INT	3
Arthropoda	Insecta	Hemiptera	*Strophingiaharteni* Hodkinson, 1981	END	1
Arthropoda	Insecta	Hemiptera	*Triozalaurisilvae* Hodkinson, 1990	NAT	9
Arthropoda	Insecta	Hymenoptera	*Bombusterrestris* (Linnaeus, 1758)	NAT	2
Arthropoda	Insecta	Hymenoptera	*Lasiusgrandis* Forel, 1909	NAT	263
Arthropoda	Insecta	Hymenoptera	*Tetramoriumcaldarium* (Roger, 1857)	INT	7
Arthropoda	Insecta	Lepidoptera	*Argyresthiaatlanticella* Rebel, 1940	END	26
Arthropoda	Insecta	Lepidoptera	*Ascotisfortunataazorica* Pinker, 1971	END	4
Arthropoda	Insecta	Lepidoptera	*Autographagamma* (Linnaeus, 1758)	NAT	1
Arthropoda	Insecta	Lepidoptera	*Cyclophoraazorensis* (Prout, 1920)	END	20
Arthropoda	Insecta	Lepidoptera	*Scopariacoecimaculalis* Warren, 1905	END	1
Arthropoda	Insecta	Neuroptera	*Hemerobiusazoricus* Tjeder, 1948	END	1
Arthropoda	Insecta	Phasmida	*Carausiusmorosus* (Sinéty, 1901)	INT	1
Arthropoda	Insecta	Psocodea	*Atlantopsocusadustus* (Hagen, 1865)	NAT	5
Arthropoda	Insecta	Psocodea	*Ectopsocusbriggsi* McLachlan, 1899	INT	17
Arthropoda	Insecta	Psocodea	*Ectopsocusstrauchi* Enderlein, 1906	NAT	6
Arthropoda	Insecta	Psocodea	*Elipsocusazoricus* Meinander, 1975	END	1
Arthropoda	Insecta	Psocodea	*Elipsocusbrincki* Badonnel, 1963	END	1
Arthropoda	Insecta	Psocodea	*Trichopsocusclarus* (Banks, 1908)	NAT	1
Arthropoda	Insecta	Psocodea	*Valenzuelaflavidus* (Stephens, 1836)	NAT	1
Arthropoda	Insecta	Thysanoptera	*Heliothripshaemorrhoidalis* (Bouché, 1833)	INT	5

**Table 4.** Inventory of birds and mammals collected in 2022 on the Protected Area for the Management of Habitats or Species of Matela, (Natural Park of Terceira Island, Azores), including taxonomical information (Kindgom, Phylum, Class, Order and Species names), colonisation status (C.S.) (END - endemic from Azores; NAT - native non-endemic; INT - introduced species; IND - indeterminate origin) and overall abundance data (N).

Phylum	Class	Order	Species	C.S.	N
Chordata	Aves	Accipitriformes	*Buteobuteorothschildi* Swann, 1919	END	6
Chordata	Aves	Charadriiformes	*Larusmichahellisatlantis* Dwight, 1922	END	4
Chordata	Aves	Columbiformes	*Columbapalumbusazorica* Hartert, E, 1905	END	92
Chordata	Aves	Passeriformes	*Erithacusrubecula* (Linnaeus, 1758)	NAT	17
Chordata	Aves	Passeriformes	*Fringillacoelebsmoreletti* Pucheran, 1859	END	128
Chordata	Aves	Passeriformes	*Motacillacinereapatriciae* Vaurie, 1957	END	24
Chordata	Aves	Passeriformes	*Oenantheoenantheleucorhoa* (Gmelin, JF, 1789)	NAT	19
Chordata	Aves	Passeriformes	*Passerdomesticus* (Linnaeus, 1758)	INT	16
Chordata	Aves	Passeriformes	*Regulusregulusinermis* Murphy & Chapin, 1929	END	6
Chordata	Aves	Passeriformes	*Serinuscanaria* (Linnaeus, 1758)	NAT	13
Chordata	Aves	Passeriformes	*Sturnusvulgarisgranti* Hartert, E, 1903	END	12
Chordata	Aves	Passeriformes	*Sylviaatricapillagularis* Alexander, 1898	END	14
Chordata	Aves	Passeriformes	*Turdusmerulaazorensis* Hartert, E, 1905	END	134
Chordata	Mammalia	Artiodactyla	*Bostaurus* Linnaeus, 1758	INT	2
Chordata	Mammalia	Carnivora	*Canislupusfamiliaris* (Linnaeus, 1758)	INT	4
Chordata	Mammalia	Carnivora	*Feliscatus* (Linnaeus, 1758)	INT	1
Chordata	Mammalia	Carnivora	*Mustelafuro* Linnaeus, 1758	INT	3
Chordata	Mammalia	Carnivora	*Mustelanivalis* Linnaeus, 1766	INT	15
Chordata	Mammalia	Chiroptera	*Nyctalusazoreum* (Thomas, 1901)	END	3
Chordata	Mammalia	Lagomorpha	*Oryctolaguscunniculus* (Linnaeus, 1758)	INT	6
Chordata	Mammalia	Rodentia	*Musmusculus* Linnaeus, 1758	INT	32
Chordata	Mammalia	Rodentia	*Rattusrattus* (Linnaeus, 1758)	INT	223
