# Supplementary material for: A multitaxa approach to biodiversity inventory in Matela protected area (Terceira, Azores, Portugal)
Source: Biodivers Data J. 2024 Apr 8;12:e121884. doi: 10.3897/BDJ.12.e121884 (PMC11019259; doi:10.3897/BDJ.12.e121884)
Supplement: Supplementary material 1 — List of bryophytes historically documented in Matela (Bryophyta, Marchantiophyta and Anthocerotophyta) [file bdj-12-e121884-s001.docx]

| **Division** | **Species** |
| --- | --- |
| **Bryophyta** | *Alophosia azorica* (Ren. & Card.) Card. |
|  | *Andoa berthelotiana* (Mont.) Ochyra |
|  | *Anomobryum julaceum* (Schrad. ex P.Gaertn., E.Mey & Scherb.) Schimp. |
|  | *Atrichum undulatum* (Hedw.) P.Beauv. |
|  | *Brachytheciastrum velutinum* (Hedw.) Ignatov & Huttunen |
|  | *Brachythecium rutabulum* (Hedw.) Schimp. |
|  | *Campylopus flexuosus* (Hedw.) Brid. |
|  | *Ceratodon purpureus* (Hedw.) Brid. subsp. purpureus |
|  | *Dicranum scottianum* Turner |
|  | *Echinodium renauldii* (Cardot) Broth. |
|  | *Exsertotheca intermedia* (Brid.) S.Olsson, Enroth & D.Quandt |
|  | *Fissidens adianthoides* Hedw. |
|  | *Fissidens asplenioides* Hedw. |
|  | *Fissidens bryoides* Hedw. |
|  | *Fissidens coacervatus* Brugg.-Nann. |
|  | *Fissidens serrulatus* Brid. |
|  | *Fissidens taxifolius* Hedw. subsp. taxifolius |
|  | *Heterocladium heteropterum* (Brid.) Schimp. |
|  | *Heterocladium wulfsbergii* I. Hagen |
|  | *Hyocomium armoricum* (Brid.) Wijk & Margad. |
|  | *Hypnum jutlandicum* Holmen & E.Warncke |
|  | *Hypnum uncinulatum* Jur. |
|  | *Isothecium prolixum* (Mitt.) M.Stech, Sim-Sim, Tangney & D.Quandt |
|  | *Kindbergia praelonga* (Hedw.) Ochyra |
|  | *Leucobryum glaucum* (Hedw.) Ångstr. |
|  | *Leucobryum juniperoideum* (Brid.) Müll. Hal. |
|  | *Mnium hornum* Hedw. |
|  | *Myurium hochstetteri* (Schimp.) Kindb. |
|  | *Oxyrrhynchium hians* (Hedw.) Loeske |
|  | *Oxyrrhynchium speciosum* (Brid.) Warnst. |
|  | *Plagiomnium undulatum* (Hedw.) T. J. Kop. |
|  | *Pseudoscleropodium purum* (Hedw.) M.Fleisch. |
|  | *Ptychomitrium polyphyllum* (Dicks. ex Sw.) Bruch & Schimp. |
|  | *Sciuro-hypnum plumosum* (Hedw.) Ignatov & Huttunen |
|  | *Sciuro-hypnum populeum* (Hedw.) Ignatov & Huttunen |
|  | *Sematophyllum substrumulosum* (Hampe) E.Britton |
|  | *Tetrastichium fontanum* (Mitt.) Cardot |
|  | *Tetrastichium virens* (Cardot) S.P.Churchill |
|  | *Thamnobryum alopecurum* (Hedw.) Gangulee |
|  | *Thamnobryum maderense* (Kindb.) Hedenäs |
|  | *Thamnobryum rudolphianum* Mastracci |
|  | *Thuidium tamariscinum* (Hedw.) Schimp. |
|  | *Tortula muralis* Hedw. |
|  | *Tortula solmsii* (Schimp.) Limpr |
|  | *Zygodon viridissimus* (Dicks.) Brid. |
| Marchantiophyta | *Blepharostoma trichophyllum* (L.) Dumort. |
|  | *Calypogeia arguta* Nees & Mont. |
|  | *Calypogeia fissa* (L.) Raddi |
|  | *Cephalozia bicuspidata* (L.) Dumort. |
|  | *Cololejeunea microscopica* (Taylor) Schiffn. |
|  | *Cololejeunea sintenisii* (Steph.) Pócs |
|  | *Conocephalum conicum* (L.) Dumort. |
|  | *Diplophyllum albicans* (L.) Dumort. |
|  | *Drepanolejeunea hamatifolia* (Hook.) Schiffn. |
|  | *Frullania azorica* Sim-Sim, Sérgio, Mues & Kraut |
|  | *Frullania fragilifolia* (Taylor) Gottsche, Lindenb. & Nees |
|  | *Frullania microphylla* (Gottsche) Pearson |
|  | *Frullania tamarisci* (L.) Dumort. |
|  | *Frullania teneriffae* (F.Weber) Nees |
|  | *Fuscocephaloziopsis crassi*folia (Lindenb. & Gottsche) Váňa & L.Söderstr. |
|  | *Harpalejeunea molleri* (Steph.) Grolle |
|  | *Heteroscyphus denticulatus* (Mitt.) Schiffn |
|  | *Lejeunea eckloniana* Lindenb. |
|  | *Lejeunea flava* (Sw.) Nees subsp. *moorei* (Lindb.) Schust. |
|  | *Lejeunea lamacerina* (Steph.) Schiffn. |
|  | *Lejeunea patens* Lindb. |
|  | *Lophocolea coadunata* (Sw.) Mont. |
|  | *Lophocolea fragrans* (Moris & De Not.) Gottsche, Lindenb. & Nees |
|  | *Marchesinia mackaii* (Hook.) Gray |
|  | *Metzgeria furcata* (L.) Corda |
|  | *Metzgeria leptoneura* Spruce |
|  | *Myriocoleopsis minutissima* (Sm.) R.L.Zhu, Y.Yu & Pócs |
|  | *Odontoschisma sphagni* (Dicks.) Dumort. |
|  | *Pellia epiphylla* (L.) Corda |
|  | *Plagiochila bifa*ria (Sw.) Lindenb. |
|  | *Plagiochila exigua* (Taylor) Taylor |
|  | *Porella canariensis* (F. Weber) Bryhn |
|  | *Porella obtusata* (Taylor) Trevis. |
|  | *Radula aquilegia* (Hook.f. & Taylor) Gottsche, Lindenb. & Nees |
|  | *Radula carringtonii* J. B. Jack |
|  | *Radula holtii* Spruce |
|  | *Radula wichurae* Steph. |
|  | *Saccogyna viticulosa* (L.) Dumort. |
|  | *Scapania gracilis* Lindb. |
|  | *Scapania nemorea* (L.) Grolle |
|  | *Solenostoma hyalinum* (Lyell) Mitt. |
|  | *Telaranea europaea* J.J.Engel & G.L.Merr. |
| **Anthocerotophyta** | *Anthoceros caucasicus* Steph. |
