# Supplementary material for: A multitaxa approach to biodiversity inventory in Matela protected area (Terceira, Azores, Portugal)
Source: Biodivers Data J. 2024 Apr 8;12:e121884. doi: 10.3897/BDJ.12.e121884 (PMC11019259; doi:10.3897/BDJ.12.e121884)
Supplement: Supplementary material 2 — List of Vascular Plants historically recorded in Matela (Lycopodiophyta, Pteridophyta, Pinophyta and Magnoliophyta) [file bdj-12-e121884-s002.docx]

| **Division** | **Species** |
| --- | --- |
| **Lycopodiophyta** | *Selaginella kraussiana* (Kunze) A. Braun |
| **Pteridophyta** | *Asplenium azoricum* (Milde) Lovis, Rasbach & Reichstein |
|  | *Asplenium scolopendrium* L. |
|  | *CuIcita macrocarpa* C. Presl |
|  | *Dryopteris aemula* (Aiton) Kuntze |
|  | *Dryopteris affinis* (Lowe) Fraser-Jenkins subsp. affinis |
|  | *Dryopteris azorica* (Christ) Alston |
|  | *Hymenophyllum tunbrigense* (L.) Sm. |
|  | *Polypodium macaronesicum* subsp. *azoricum* (Vasc.) Rumsey, Carine & Robba |
|  | *Pteridium aquilinum* (L.) Kuhn |
|  | *Pteris incompleta* Cav. |
|  | *Sphaeropteris cooperi* (F. Muell.) R.M.Tryon |
|  | *Struthiopteris spicant* (L.) Weis |
|  | *Vandenboschia speciosa* (Willd.) G.Kunkel |
| **Pinophyta** | Juniperus brevifolia (Hochst. ex Seub.) Antoine subsp*. brevifolia* |
| **Magnoliophyta** | *Acacia melanoxylon* R. Br. |
|  | *Calluna vulgaris* (L.) Hull |
|  | *Erica azorica* Hochst. ex Seub. |
|  | *Eucalyptus globulus* Labill. |
|  | *Frangula azorica* Grubov |
|  | *Hedera azorica* Carrière |
|  | *Hedychium gardneranum* Sheppard ex Ker Gawl. |
|  | *Holcus rigidus* Hochst. ex Seub. |
|  | *Ilex azorica* Gand. |
|  | *Juncus effusus* L. |
|  | *Laurus azorica* (Seub.) Franco |
|  | *Leycesteria formosa* Wall. |
|  | *Lysimachia azorica* Hornem. ex Hook. |
|  | *Marrubium vulgare* L. |
|  | *Morella faya* (Aiton) Wilbur |
|  | *Phytolacca americana* L. |
|  | *Picconia azorica* (Tutin) Knobl. |
|  | *Pittosporum undulatum* Vent. |
|  | *Potentilla anglica* Laichrd. |
|  | *Potentilla indica* (Andrews) T.Wolf |
|  | *Rubia agostinhoi* Dansereau & P. Silva |
|  | *Rubus ulmifolius* Schott |
|  | *Rumex* sp. |
|  | *Solanum mauritianum* Scop. |
|  | *Ulex europaeus* L. subsp. *europaeus* |
|  | *Vaccinium cylindraceum* Sm. |
|  | *Vinca difformis* Pourr. |
