# Supplementary material for: A multitaxa approach to biodiversity inventory in Matela protected area (Terceira, Azores, Portugal)
Source: Biodivers Data J. 2024 Apr 8;12:e121884. doi: 10.3897/BDJ.12.e121884 (PMC11019259; doi:10.3897/BDJ.12.e121884)
Supplement: Supplementary material 3 — List of arthropods historically documented in Matela (Arthropoda) [file bdj-12-e121884-s003.docx]

| **PHYLLUM** | **Species** |
| --- | --- |
| **Arthropoda** | *Acorigone acoreensis* (Wunderlich, 1992) |
|  | *Agalenatea redii* (Scopoli, 1763) |
|  | *Agyneta fuscipalpa* (C. L. Koch, 1836) |
|  | *Aleochara bipustulata* (Linnaeus, 1760) |
|  | *Aloconota sulcifrons* (Stephens, 1832) |
|  | *Anaspis proteus* Wollaston, 1854 |
|  | *Anotylus nitidifrons* (Wollaston, 1871) |
|  | *Anthocoris nemoralis* (Fabricius, 1794) |
|  | *Aphis aurantii* Boyer de Fonscolombe, 1841 |
|  | *Aphrodes hamiltoni* Quartau & Borges, 2003 |
|  | *Argyresthia atlanticella* Rebel, 1940 |
|  | *Ascotis fortunata azorica* Pinker, 1971 |
|  | *Atheta aeneicollis* (Sharp, 1869) |
|  | *Atheta fungi* (Gravenhorst, 1806) |
|  | *Athous azoricus* Platia & Gudenzi, 2002 |
|  | *Atlantocis gillerforsi* Israelson, 1986 |
|  | *Atlantopsocus adustus* (Hagen, 1865) |
|  | *Bertkauia lucifuga* (Rambur, 1842) |
|  | *Blaniulus guttulatus* (Fabricius, 1798) |
|  | *Brachysteles parvicornis* (A. Costa, 1847) |
|  | *Calacalles subcarinatus* (Israelson, 1984) |
|  | *Canariphantes acoreensis* (Wunderlich, 1992) |
|  | *Catops coracinus* Kellner, 1846 |
|  | *Cheiracanthium erraticum* (Walckenaer, 1802) |
|  | *Cheiracanthium mildei* L. Koch, 1864 |
|  | *Choneiulus palmatus* (Nemec, 1895) |
|  | *Chrysodeixis chalcites* (Esper, 1789) |
|  | *Chthonius ischnocheles* (Hermann, 1804) |
|  | *Cinara juniperi* (De Geer, 1773) |
|  | *Cixius azoterceirae* Remane & Asche, 1979 |
|  | *Clubiona terrestris* Westring, 1851 |
|  | *Coccotrypes carpophagus* (Hornung, 1842) |
|  | *Cordalia obscura* (Gravenhorst, 1802) |
|  | *Cryptachaea blattea* (Urquhart, 1886) |
|  | *Cyclophora azorensis* (Prout, 1920) |
|  | *Cylindroiulus propinquus* (Porat, 1870) |
|  | *Cyphopterum adcendens* (Herrich-Schäffer, 1835) |
|  | *Damaeus pomboi* Pérez-Íñigo, 1987 |
|  | *Dilta saxicola* (Womersley, 1930) |
|  | *Drouetius borgesi borgesi* Machado, 2009 |
|  | *Dysdera crocata* C.L. Koch, 1838 |
|  | *Ectopsocus briggsi* McLachlan, 1899 |
|  | *Ectopsocus strauchi* Enderlein, 1906 |
|  | *Elipsocus azoricus* Meinander, 1975 |
|  | *Elipsocus brincki* Badonnel, 1963 |
|  | *Emblyna acoreensis* Wunderlich, 1992 |
|  | *Empicoris rubromaculatus* (Blackburn, 1889) |
|  | *Ephippiochthonius tetrachelatus* (Preyssler, 1790) |
|  | *Epuraea biguttata* (Thunberg, 1784) |
|  | *Erigone atra* Blackwall, 1833 |
|  | *Erigone dentipalpis* (Wider, 1834) |
|  | *Ero furcata* (Villers 1789) |
|  | *Euborellia annulipes* (Lucas, 1847) |
|  | *Eupteryx azorica* Ribaut, 1941 |
|  | *Galumna azoreana* Pérez-Íñigo, 1992 |
|  | *Gibbaranea occidentalis* Wunderlich, 1989 |
|  | *Heliothrips haemorrhoidalis* (Bouché) |
|  | *Hermanniella incondita* Pérez-Íñigo, 1987 |
|  | *Heterotoma planicornis* (Pallas, 1772) |
|  | *Homalenotus coriaceus* (Simon, 1879) |
|  | *Hoplothrips corticis* (De Geer, 1773) |
|  | *Humerobates pomboi* Pérez-Íñigo, 1992 |
|  | *Kleidocerys ericae* (Horváth, 1909) |
|  | *Lasaeola oceanica* Simon, 1883 |
|  | *Lasius grandis* Forel, 1909 |
|  | *Lathys dentichelis* (Simon 1883) |
|  | *Leiobunum blackwalli* Meade, 1861 |
|  | *Lithobius pilicornis pilicornis* Newport, 1844 |
|  | *Macaroeris cata* (Blackwall, 1867) |
|  | *Macaroeris diligens* (Blackwall, 1867) |
|  | *Mangora acalypha* (Walckenaer, 1802) |
|  | *Mecinus pascuorum* (Gyllenhal, 1813) |
|  | *Megamelodes quadrimaculatus* (Signoret, 1865) |
|  | *Metellina merianae* (Scopoli, 1763) |
|  | *Microlinyphia johnsoni* (Blackwall, 1859) |
|  | *Monalocoris filicis* (Linnaeus, 1758) |
|  | *Monomorium carbonarium* (Smith, 1858) |
|  | *Mythimna unipuncta* (Haworth, 1809) |
|  | *Nabis pseudoferus ibericus* Remane, 1962 |
|  | *Nigma puella* (Simon, 1870) |
|  | *Nothrus palustris azorensis* Pérez-Íñigo, 1992 |
|  | *Ocypus aethiops* (Waltl, 1835) |
|  | *Oedothorax fuscus* (Blackwall, 1834) |
|  | *Ommatocepheus parvilamellatus* Pérez-Íñigo & Pérez-Íñigo Jr., 1996 |
|  | *Ommatoiulus moreleti* (Lucas, 1860) |
|  | *Orthochaetes insignis* (Aubé, 1863) |
|  | *Orthonama obstipata* (Fabricius, 1794) |
|  | *Oxidus gracilis* (C.L. Koch, 1847) |
|  | *Paraphloeostiba gayndahensis* (MacLeay, 1871) |
|  | *Pardosa acorensis* Simon, 1883 |
|  | *Peripsocus phaeopterus* (Stephens, 1836) |
|  | *Phthiracarus atlanticus* (Pérez-Íñigo, 1987) |
|  | *Pinalitus oromii* J. Ribes, 1992 |
|  | *Pisaura acoreensis* Wunderlich, 1992 |
|  | *Plinthisus minutissimus* Fieber, 1864 |
|  | *Polydesmus coriaceus* Porat, 1870 |
|  | *Popillia japonica* Newman, 1838 |
|  | *Porrhoclubiona decora* (Blackwall, 1859) |
|  | *Porrhoclubiona genevensis* (L. Koch, 1866) |
|  | *Proteinus atomarius* Erichson, 1840 |
|  | *Pseudechinosoma nodosum* Hustache, 1936 |
|  | *Pseudophloeophagus tenax borgesi* Stüben, 2022 |
|  | *Pterostichus aterrimus aterrimus* (Herbst, 1784) |
|  | *Rhopalomesites tardyi* (Curtis, 1825) |
|  | *Rhopobota naevana* (Hübner, 1817) |
|  | *Rugathodes acoreensis* Wunderlich, 1992 |
|  | *Savigniorrhipis acoreensis* Wunderlich, 1992 |
|  | *Scolopostethus decoratus* (Hahn, 1833) |
|  | *Scoparia coecimaculalis* Warren, 1905 |
|  | *Sericoderus lateralis* (Gyllenhal, 1827) |
|  | *Steatoda grossa* (C.L. Koch, 1838) |
|  | *Steganacarus hirsutus azorensis* Pérez-Íñigo, 1992 |
|  | *Stelidota geminata* (Say, 1825) |
|  | *Strophingia harteni* Hodkinson, 1981 |
|  | *Tachyporus nitidulus* (Fabricius, 1781) |
|  | *Tegenaria domestica* (Clerck, 1757) |
|  | *Temnothorax unifasciatus* (Latreille, 1798) |
|  | *Tenuiphantes miguelensis* (Wunderlich, 1992) |
|  | *Tenuiphantes tenuis* (Blackwall, 1852) |
|  | *Theridion musivivum* Schmidt, 1956 |
|  | *Trichadenotecnum castum* Betz, 1983 |
|  | *Trichopsocus clarus* (Banks, 1908) |
|  | *Trigoniophthalmus borgesi* Mendes, Gaju, Bach & Molero, 2000 |
|  | *Trioza laurisilvae* Hodkinson, 1990 |
|  | *Valenzuela flavidus* (Stephens, 1836) |
|  | *Xanthorhoe inaequata* Warren, 1905 |
|  | Xyleborinus alni Nijima, 1909 |
|  | *Zetha simonyi* (Krauss, 1892) |
