# Supplementary material for: A multitaxa approach to biodiversity inventory in Matela protected area (Terceira, Azores, Portugal)
Source: Biodivers Data J. 2024 Apr 8;12:e121884. doi: 10.3897/BDJ.12.e121884 (PMC11019259; doi:10.3897/BDJ.12.e121884)
Supplement: Supplementary material 4 — List of vertebrates historically documented in Matela [file bdj-12-e121884-s004.docx]

| **PHYLLUM** | **Species** |
| --- | --- |
| **Chordata** | *Buteo buteo rothschildi* (Swann, 1919) |
|  | *Columba palumbus azorica* Hartert, 1905 |
|  | *Erithacus rubecula rubecula* (Linnaeus, 1758) |
|  | *Fringilla coelebs moreletti* Pucheran, 1859 |
|  | *Motacilla cinerea patriciae* Vaurie, 1957 |
|  | *Nyctalus azoreum* (Thomas, 1901) |
|  | *Passer domesticus domesticus* (Linnaeus, 1758) |
|  | *Regulus regulus inermis* (Murphy & Chapin, 1929) |
|  | *Sturnus vulgaris granti* Hartert, 1903 |
|  | *Sylvia atricapilla guralis* Alexander, 1898 |
|  | *Turdus merula azorensis* Hartert, 1905 |
