# Supplementary material for: A multitaxa approach to biodiversity inventory in Matela protected area (Terceira, Azores, Portugal)
Source: Biodivers Data J. 2024 Apr 8;12:e121884. doi: 10.3897/BDJ.12.e121884 (PMC11019259; doi:10.3897/BDJ.12.e121884)
Supplement: Supplementary material 5 — List of historical literature sources mentioning Matela [file bdj-12-e121884-s005.docx]

Aparício, B. M. A. (2016). *Climate Change implications on landscape functional connectivity: a case study with insects in Terceira Island, Azores* [Master's thesis, Universidade de Lisboa]. Repositório da Universidade de Lisboa.

Aptroot, A., & Rodrigues, A. F. (2005). Additions to the Azorean lichen Flora. *Arquipélago. Life and Marine Sciences,* 22A, 71-75.

Aranda, S. C., Hespanhol, H., Homem, N., Borges, P. A., Lobo, J. M., & Gabriel, R. (2015). The iterative process of plant species inventorying for obtaining reliable biodiversity patterns. *Botanical Journal of the Linnean Society*, 177(4), 491-503.

Bates, J. W., & Gabriel, R. (1997). *Sphagnum cuspidatum* and *S. imbricatum* ssp. *affine* new to Macaronesia, and other new island records for Terceira, Azores. *Journal of Bryology*, 19(3), 645-648.

Borges, P. A., Aguiar, C., Amaral, J., Amorim, I. R., André, G., Arraiol, A., Baz, A., Dinis, F., Enghoff, H., Gaspar, C., Ilharcoo, F., Mahnert, V., Melo, C., Perreira, F., Quartau, J., A., Ribeiro, S., P., Ribes, J., Serrano, A., R., M., Sousa, A., B., Strassen, R., Z., Vieira, L., Vieira, V., Vitorino, A., & Wunderlich, J. (2005). Ranking protected areas in the Azores using standardised sampling of soil epigean arthropods. *Biodiversity & Conservation*, 14(9), 2029-2060.

Borges, P. A., Gaspar, C., Santos, A., Ribeiro, S. P., Cardoso, P., Triantis, K. A., & Amorim, I. R. (2011). Patterns of colonization and species distribution for Azorean arthropods: evolution, diversity, rarity and extinction. *Açoreana: Revista de Estudos Açoreanos*, 7, 93-123.

Borges, P. A., Hortal, J., Gabriel, R., & Homem, N. (2009). Would species richness estimators change the observed species area relationship?. *Acta Oecologica*, 35(1), 149-156.

Borges, P. A., Lobo, J. M., de Azevedo, E. B., Gaspar, C. S., Melo, C., & Nunes, L. V. (2006). Invasibility and species richness of island endemic arthropods: a general model of endemic vs. exotic species. Journal of Biogeography, 33: 169-187.

Caniaux, G. (2013). Les ignimbrites des Açores. *Açoreana: Revista de Estudos Açoreanos*, 10(4), 619-644.

Cardoso, P., Borges, P. A., & Gaspar, C. (2006). Biotic integrity of the arthropod communities in the natural forests of Azores. *Biodiversity and Conservation*, 16(10), 2883-2901.

Clerc, P. (2006). Synopsis of Usnea (lichenized Ascomycetes) from the Azores with additional information on the species in Macaronesia. *The Lichenologist*, 38(3), 191-212.

CMAH. (2007). *Restauro ecológico da Área Protegida da Matela*. Angra do Heroísmo

CMAH. (2015). *Carta educativa de Angra do Heroísmo*. Angra do Heroísmo.

Coelho, R. M. L. (2015). *Plano de gestão e conservação de* Azorina vidalii (Wats.) Feer. [Master's thesis, Universidade dos Açores]. Repositório da Universidade dos Açores.

Corvelo, R. A. F. (2010). *Estatuto de conservação das plantas vasculares endémicas dos Açores segundo os critérios da IUCN: implicações ao nível do ordenamento do território e do planeamento ambiental* [Master's thesis, Universidade dos Açores]. Repositório da Universidade dos Açores.

Couto, A. B. (2010). *Padrões de distribuição dos briófitos dos Açores em diferentes escalas: Contributo para a conservação de espécies ameaçadas* [Master's thesis, Universidade dos Açores]. Repositório da Universidade dos Açores.

Dias, E. (1996). *Vegetação Natural Dos Açores: Ecologia e Sintaxonomia das Florestas Naturais* [Doctoral dissertation, Universidade dos Açores]. Repositório da Universidade dos Açores.

Elias, R. B., & Dias, E. (2014). The recognition of infraspecific taxa in Juniperus brevifolia (Cupressaceae). *Phytotaxa*, 188(5), 241-250.

Gabriel, R. (1994). *Briófitos da Ilha Terceira (Açores). Ecologia, distribuição e vulnerabilidade de espécies seleccionadas* – APCC. Departamento de Ciências Agrárias. Universidade dos Açores. Angra do Heroísmo.

Gabriel, R., & Bates, J. W. (2005). Bryophyte community composition and habitat specificity in the natural forests of Terceira, Azores. *Plant ecology*, 177(1), 125-144.

Gertisser, R., Self, S., Gaspar, J. L., Kelley, S. P., Pimentel, A., Eikenberg, J., Barry, T. L., Pacheco, J. M. & Vespa, M. (2010). Ignimbrite stratigraphy and chronology on Terceira Island, Azores. *The Geological Society of America, Special Paper*, 464, 133-154.

Goulart, H. F. S. (2015). *Plantas vasculares invasoras no Parque Natural da Ilha Terceira: caracterização e monitorização do controlo de* Pittosporum undulatum. [Master's thesis, Universidade dos Açores]. Repositório da Universidade dos Açores.

Hortal, J., Borges, P. A., & Gaspar, C. (2006). Evaluating the performance of species richness estimators: sensitivity to sample grain size. *Journal of animal ecology*, 75(1), 274-287.

Jeffery, A. J., Gertisser, R., Self, S., Pimentel, A., O’Driscoll, B., & Pacheco, J. M. (2017). Petrogenesis of the peralkaline ignimbrites of Terceira, Azores. *Journal of Petrology*, 58(12), 2365-2402.

Leal, A. C. A. (2011). *Paul da pedreira do Cabo da Praia: contributo das percepções ambientais para a sua preservação* [Master's thesis, Universidade dos Açores]. Repositório da Universidade dos Açores.

Lamelas López, L. (2016). *Cambios antrópicos y variación espacio-temporal en comunidades de macroinvertebrados acuáticos de lagunas oceánicas: el caso del archipiélago de las Azores*. Tese de Mestrado em Gestão e Conservação da Natureza, Universidade dos Açores, Angra do Heroísmo. Portugal. [Master's thesis, Universidade dos Açores]. Repositório da Universidade dos Açores.

Machado, A. (2009). El género *Drouetius* Méquignon, 1942 stat. prom., de las islas Azores (Coleoptera, Curculionidae, Entiminae). *Graellsia*, 65(1), 19-46.

Machado, P. L. A. S. (2019). *Estudo paleoclimático e paleobotânico de Angra do Heroísmo a partir dos fósseis incorporados nas cinzas vulcânicas do Monte Brasil* [Master's thesis, Universidade dos Açores]. Reportório da Universidade dos Açores.

Pimentel, A. H. G. (2016). *Pyroclastic density current-forming eruptions on Faial and Terceira Islands, Azores*. [Doctoral dissertation, Universidade dos Açores]. Repositório da Universidade dos Açores.

Pimentel, A., Self, S., Pacheco, J. M., Jeffery, A. J., & Gertisser, R. (2021). Eruption style, emplacement dynamics and geometry of peralkaline ignimbrites: Insights from the Lajes-Angra ignimbrite formation, Terceira Island, Azores. *Frontiers in Earth Science*, 9, 503.

Platia, G. & Borges, P.A.V. (2002). Description of a new species of *Athous* and record of the female of *A. azoricus* Platia & Gudenzi from the Azores (Coleoptera, Elateridae). *Elytron*, 16, 91-95.

Purvis, O. W., James, P. W., Smith, C. W., & Dias, E. (1998). Studies in the lichens of the Azores. Part 5: a comparison between the lichen floras associated with *Juniperus brevifolia* in relict woodland in selected sites on Terceira and Flores. *Boletim do Museu Municipal do Funchal*, 5, 333-348.

Rego, C., Boieiro, M., Vieira, V., & Borges, P. A. (2015). The biodiversity of terrestrial arthropods in Azores. *Revista IDE@-SEA*, 5B, 1-24.

Ribeiro, S. P., Borges, P. A., Gaspar, C., Melo, C., Serrano, A. R., Amaral, J., Aguiar, C., Anfré, G. & Quartau, J. A. (2005). Canopy insect herbivores in the Azorean Laurisilva forests: key host plant species in a highly generalist insect community. *Ecography*, 28(3), 315-330.

Ribeiro, S.P. & Borges, P.A.V. (2010). Canopy habitat area effect on the arthropod species densities in the Azores: pondering the contribution of tourist species and other life histories. In A.R.M. Serrano, P.A.V. Borges, M. Boieiro & P. Oromí (Eds.), Terrestrial arthropods of Macaronesia – Biodiversity, Ecology and Evolution. Sociedade Portuguesa de Entomologia (pp. 89-114). Sociedade Portuguesa de Entomologia.

Schumm, F., & Aptroot, A. (2013). *Flechten Madeiras, der Kanaren und Azoren-Band* 2 Ergänzungsband. BoD–Books on Demand.

Stüben, P. E., & Borges, P. A. (2019). Die Curculionoidea (Coleoptera) von den Inseln der Azoren. *SNUDEBILLER: Studies on taxonomy, biology and ecology of Curculionoidea*, 20 (279), 59.

Szaszkiewicz, S., S. (2019). Análisis de la gestión de los Espacios Naturales Protegidos en las Azores. [Final Course Project, Universidade de Cádiz]. Repositório Institucional da Universidade de Cádiz.

Telleria, M. T., Melo, I., Dueñas, M., Larsson, K. H., & Paz Martín, M. P. (2013). Molecular analyses confirm Brevicellicium in Trechisporales. *International Mycological Association Fungus*, 4(1), 21-28.

Telleria, M. T., Melo, I., Duenas, M., Rodriguez-Armas, J. L., Beltran-Tejera, E., Cardoso, J., & Salcedo, I. (2009). Diversity and richness of corticioid fungi (Basidiomycota) on Azores Islands: a preliminary survey. *Nova Hedwigia*, 88(3), 285.
